# Overcoming Extreme Ammonia Inhibition on Methanogenesis by Artificially Constructing a Synergistically Community with Acidogenic Bacteria and Hydrogenotrophic Archaea

**DOI:** 10.1002/advs.202502743

**Published:** 2025-03-31

**Authors:** Heng Wu, Huaiwen Zhang, Taili Dong, Zhenyu Li, Xiaohui Guo, Heyu Chen, Yiqing Yao

**Affiliations:** ^1^ College of Mechanical and Electronic Engineering Northwest A&F University Yangling Shaanxi 712100 P.R. China; ^2^ Shandong Min‐he Biotechnology Co. Ltd. Penglai 265600 China; ^3^ Water Technologies Innovation Institute & Research Advancement Saudi Water Authority P.O. Box 8328 Al‐Jubail 31951 Saudi Arabia

**Keywords:** ammonia nitrogen inhibition, ammonia nitrogen tolerance inoculum, engineering application, hydrolysis pretreatment, microbial mechanism

## Abstract

High total ammonia nitrogen (TAN) inhibits anaerobic digestion (AD) and cannot be completely eliminated by merely enhancing a stage of AD. This study incorporates TAN‐tolerant inoculum into substrates hydrolyzed by Rhizopus mixed agents to simultaneously enhance hydrolysis‐acidogenesis‐methanogenesis. The results show a 16.46‐fold increase in CH4 production under TAN‐inhibited (6870.97 mg L−1) conditions, even exceeding the AD without TAN by 21.10%. Model substrates sodium acetate and mixed H2 confirm hydrogenotrophic methanogenesis is the main pathway, with reduced TAN inhibition. Furthermore, a synergistic metabolic microbial community dominated by hydrolytic bacteria JAAYGG01 sp. and DTU014 sp., acidogenic bacteria DTU015 sp., DTU013 sp., and JAAYLO01 sp., and methanogens Methanosarcina mazei and an unclassified species in the Methanoculleus is reconstructed to resist TAN inhibition. Metagenomic combined with metatranscriptomic sequencing identifies that this microbial community carries xynD and bglB to regulate substrate hydrolysis, leading to acetate production through glycolysis, butyrate, and pyruvate metabolism with high acetate kinase activity, thereby CH4 produced primarily via hydrogenotrophic methanogenesis with high coenzyme F420 activity, facilitated by efficient mass transfer processes and quorum sensing regulation. This cleaner strategy obtains higher economic benefit (US$149.02) than conventional AD and can increase 154.64‐fold energy production of a 24 000 m3 biogas plant, guided by machine learning.

## Graphical abstract

This study proposes a two‐step biological strategy to synergistically enhance the three stages of anaerobic digestion, ultimately eliminating the extreme ammonia inhibition and breaking through the inefficiency bottleneck of conventional biotechnology. Mechanism analysis reveals the key pathways of hydrolysis, acidogenesis, and methanogenesis. Application potential analysis indicates that this strategy is clean, safe, and highly economically beneficial.

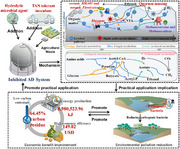

## 1 Introduction

The rapid development of livestock production has increased livestock manure (LM) discharge, totaling over 400 Mt tons annually worldwide.^[^ ^1^ ^]^ Although the discharge of untreated LM will damage the environment seriously,^[^ ^2^ ^]^ it essentially represents a potential resource with cyclic utilization.^[^ ^3^ ^]^ Anaerobic digestion (AD) is a widely used and effective measure for recovering resources from LM for methane (CH_4_) production.^[^ ^4^ ^]^ However, during the AD process, LM is prone to releasing high concentrations of total ammonia nitrogen (TAN),^[^ ^5^ ^]^ which inhibits CH_4_ production per unit of volatile solid (VS) and leads to the accumulation of volatile fatty acids (VFAs), ultimately leading to AD failure. Although the membrane contactor and electrochemical cell can remove 70% and 61.90% nitrogen concentration, respectively,^[^ ^6^, ^7^ ^]^ but will undoubtedly increase the high cost. In comparison, biological methods have received widespread attention due to their clean and low‐cost characteristics.^[^ ^8^ ^]^

It is well known that methanogenesis is the step most severely inhibited by TAN.^[^ ^9^ ^]^ It is worth noting that AD residues are rich in high‐abundance methanogens that are tolerant to low concentrations of TAN,^[^ ^10^, ^11^ ^]^ holding the potential to be used as bioaugmentation agents. By gradually increasing the tolerance of these methanogens to TAN, introducing them into the AD system will achieve rapid methanogens enrichment and methanogenic pathways under TAN concentration conditions,^[^ ^12^ ^]^ thereby alleviating inhibition on methanogenesis. This approach is not only efficient but also easy to operate and suitable for large‐scale practical applications.

Nevertheless, the AD process relies on the synergistic metabolism of bacteria and archaea.^[^ ^13^ ^]^ Focusing only on methanogen enrichment while neglecting the bioaugmentation of hydrolytic and acidogenic microbial communities would hinder the complete alleviation of TAN inhibition. However, anaerobic functional microorganisms with efficient hydrolysis performance are rarely reported, and their large‐scale cultivation is more difficult. It should be noted that the fungus strain *Rhizopus arrhizus* secretes hydrolases to degrade polysaccharides,^[^ ^14^ ^]^ thereby holding potential for enhancing the hydrolysis, along with efficient acidogenesis. During this process, the efficient hydrolytic and acidogenic bacterial communities were reshaped,^[^ ^15^, ^16^ ^]^ contributing to long‐term and sustained hydrolysis and acidogenesis. Importantly, *R. arrhizus* mixed agent (RMA) has been mass‐produced and marketed at low cost and readily available.^[^ ^14^ ^]^ In theory, the inhibitory effect of TAN can be eliminated by using a RMA to promote hydrolysis and acidogenicity based on AD residue inoculation.

Based on the above, we used representative LM cow dung (CD) as a substrate and inoculated with waste activated sludge (WAS) for co‐AD. The following measures were taken to synergistically eliminate the TAN inhibition of AD system: (1) First, the AD residues with a tolerance to TAN of about 6000 mg L^−1^ were obtained by gradient domestication, which was used for methanogenesis bioaugmentation inocula. (2) Second, the AD system with TAN inhibition was subjected to a 2‐d hydrolysis pretreatment using RMA, enhancing hydrolysis and acidogenesis. (3) Third, the AD residues obtained in (1) were inoculated to the AD system after hydrolysis pretreatment obtained in (2). After constructing this AD system, we investigated: (1) if the hydrolysis pretreatment coupled with methanogenic enhancement can completely alleviate TAN inhibition, and identified the key main methanogenic pathways; (2) if metagenomics coupled with metatranscriptome could be used to reveal the multi‐pathway synergistic enhancement mechanism of hydrolysis‐acidogenesis‐methanogenesis; and (3) systematically the consequent life cycle impacts and economic benefits, using machine learning models to optimize application effectiveness. To the best of our knowledge, this work is the first to use two easy‐to‐operate biotechnology to achieve complete elimination of ultrahigh TAN concentration inhibition, providing novel strategies and insights for increasing methane production in practical biogas plant with high TAN loads.

## 2 Results

### 2.1 Enhancement of Entire Anaerobic Digestion Performance

**Figure 1:**
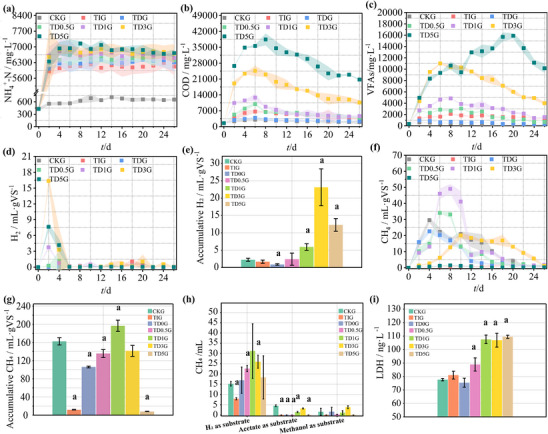
a) NH_4_^+^‐N concentration, b) COD concentration, c) VFA concentration, d) daily H_2_ production, e) cumulative H_2_ production, f) daily CH_4_ production, g) cumulative CH_4_ production, h) validation of key methanogenic pathways using different substrates, and i) LDH concentration under different AD conditions. The width of the color ribbon represents the error interval. CK was used for *t*‐test analysis with other experimental groups, and marker a indicated significant differences (*P* < 0.05).

These AD systems with TAN underwent hydrolysis pretreatment with 0, 0.5, 1, 3, and 5 g RMA/g TS, corresponding to TD0G, TD0.5G, TD1G, TD3G, and TD5G, respectively, and adding TAN‐tolerant inoculum to compare with the control check group (CKG) without TAN and the TAN‐inhibited group (TIG). The average concentrations of NH_4_^+^‐N (**Figures** 1a and S1a, Supporting Information) and free ammonia (Figure S1a–c, Supporting Information) in CK are only 637.79 and 17.89 mg L^−1^, which was significantly (*P* < 0.05, Figure S1a–c, Supporting Information) lower than that in the other AD systems, which ranged from 6119.17 to 6846.13 mg L^−1^ for NH_4_^+^‐N and 75.31 to 279.52 mg L^−1^ for free ammonia.

Due to TAN inhibition in the utilization of organic matter, the chemical oxygen demand (COD) concentration of TIG was significantly higher (*P* < 0.05, Figure S1d, Supporting Information) than that of CKG (Figure 1b). After inoculation of TAN‐tolerant inocula, the COD concentration of TD0G was lower than that of TIG. It should be noted that the continuous significant increase (*P* < 0.05, Figure S1d, Supporting Information) of COD peaks (1.50–5.82 times more than TIG) with the increase of RMA addition ratio in TD0.5G to TD5G. The increased electrical conductivity (EC) value from RMA addition also confirmed the promoted hydrolysis (Figure S1e,f, Supporting Information).

In addition, VFA concentration in TIG was significantly (*P* < 0.05, Figure S1g, Supporting Information) higher than that of CKG because it was not consumed (Figure 1c). Inoculants alleviated TAN inhibition on methanogenesis, resulting in a decrease in VFA concentration in TD0G compared to TIG. In addition, as the proportion of RMA increased in TD0.5G to TD5G, the peak VFAs significantly increased (*P* < 0.05, Figure S1g, Supporting Information) from 3046.73 to 15 888.21 mg L^−1^, and their acetate proportion was 1.24–1.47 times higher than TIG (Figure S1h–n, Supporting Information). The high fluctuation of ethanol concentration in TDG may also improve the CH_4_ production (Figure S1o,p, Supporting Information).

In addition, the rapid hydrolysis and acidogenesis resulted in significantly higher (*P* < 0.05, Figure S1q, Supporting Information) hydrogen (H_2_) production (Figure 1d,e), which helped to strengthen the hydrogenotrophic methanogenesis. High VFA concentration hindered methanogenesis (Figure 1f,g) in TD3G and TD5G, leading to CH_4_ production of TDIG of 196.71 mL/gVS, which is 16.9 times that of TIG (11.95 mL/gVS) and even significantly higher (*P* < 0.05, Figure S1r, Supporting Information) than CKG (162.43 mL/gVS). This study demonstrates a substantial advantage in boosting CH_4_ production over standard biochar and electrochemical methods (2.04–2.20 times improvement).^[^ ^17^, ^18^ ^]^ As a verification, the CH_4_ production of the TD1G‐inactivation was significantly lower (*P* < 0.05, Figure S2a,b, Supporting Information) than that of TD1G and CKG. This confirms that the reason for overcoming TAN inhibition was not through using RMA itself as a substrate, but rather through the metabolic activity of the RMA that promotes hydrolysis and VFA production.

In TD1G, it has been verified that its hydrolytic and acidogenic potential was significantly higher than those in TIG and CKG (Figure S3, Supporting Information). More importantly, its key methanogenic pathway was verified. The hydrogenotrophic methanogenesis pathway contributes more significantly to the CH_4_ production, accounting for about 93%, and its methanogenic potential is notably stronger than that of CKG and TIG (Figure 1h). After inoculation with TAN‐tolerant inoculum, the microbial activity was improved, which was the premise to ensure the CH_4_ production (Figure 1i). Due to introduction of RMA along with continuous hydrolysis, lactate dehydrogenase (LDH) concentration increased subsequently (Figure 1i).

Therefore, 1 g/g total solid (TS) RMA hydrolysis pretreatment and TAN‐tolerant inoculum, namely TD1G, enhanced the entire AD process resisting 6870.97 TAN mg/ L^−1^ for higher CH_4_ production than TIG, even surpassing CKG, overcoming the technical bottleneck caused by extreme inhibition conditions.

### 2.2 Efficient Synergistic Reconstruction of Microbial Community

In TD1G, the alpha diversity of bacterial and archaeal communities changed (Tables S1 and S2, Supporting Information). Principal component analysis and redundancy analysis confirmed that the bacterial community was reconstructed due to the addition of RMA, while the archaeal community was mainly affected by the concentration of RMA and TAN (Figure S4a–d, Supporting Information), ultimately leading to significant differences in TD1G compared to TIG (Figure S5a,b, Supporting Information).

#### 2.2.1 Further Identification of Key Hydrolytic Fungal Genera

The RMA consists of microorganisms adapted to different temperatures. To maximize its hydrolytic potential and promote rapid colonization, the temperature is initially set at 35 °C to facilitate rapid hydrolysis by the *Rhizopus* ^[^ ^14^, ^19^ ^]^ and *Mucor* ^[^ ^20^ ^]^ (Table S3, Supporting Information). When the temperature is raised to 55 °C, environmental stress favors the continuous enrichment and dominance of thermotolerant *Rhizopus* (79.80%)^[^ ^14^, ^19^ ^]^ after colonization in TD1G (Figure S6, Supporting Information), replacing the indigenous microorganisms *Xylaria* ^[^ ^21^ ^]^ (31.99%), *Racocetra* ^[^ ^22^ ^]^ (10.88%) in CKG, and *Xylaria* (40.01%), *Aspergillus* (1.91%)^[^ ^23^ ^]^ in TIG, thereby continuously enhancing hydrolysis.

**Figure 2:**
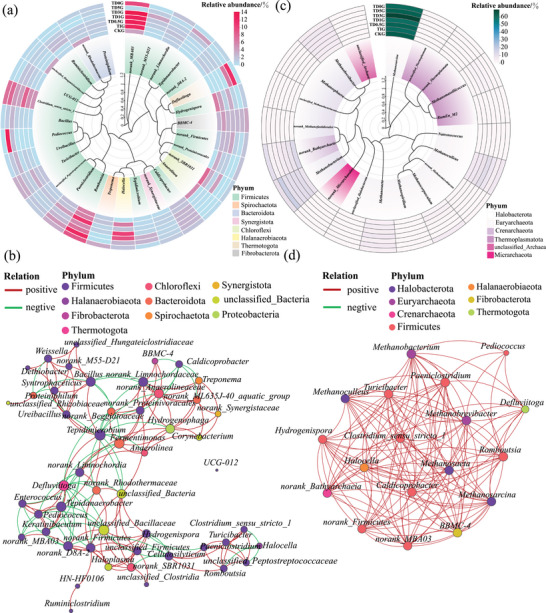
a) Unrooted developmental trees based on the maximum likelihood method and relative abundance of bacteria under different AD conditions and b) its coexistence network based on Pearson correlation (*P* < 0.05). c) Unrooted developmental trees based on the maximum likelihood method and relative abundance of archaea under different AD conditions and d) its coexistence network with bacteria based on Pearson correlation (*P* < 0.05). Significance testing was done by *t‐*test: ****P < 0.0001; ***P < 0.001; **P < 0.01; and *P < 0.05.

Rapid hydrolysis and acidogenesis drove the decrease in pH (Figure S7a, Supporting Information), which in turn led to the reconstruction of hydrolytic and acidogenic bacterial communities (Figure S7b, Supporting Information), and the key genera have been identified (**Figure** **2**a). Under high‐concentration TAN inhibition in TIG, the primary enriched genera were the stress‐resistant unranked SBR1031,^[^ ^24^ ^]^ unranked MBA03^[^ ^25^ ^]^ (12.48%), and unranked Firmicutes^[^ ^26^ ^]^ (2.64%). Due to the abundance of *Romboutsia* ^[^ ^27^ ^]^ in the TAN‐tolerant inoculum, the dominant genera in TD0G shifted to unranked MBA03 (9.63%) and *Romboutsia* (8.90%). With an increasing RMA ratio, unranked MBA03 and unranked Firmicutes were driven to enrich up to 13.12% and 3.84% in TD1G, respectively, confirming their roles as key genera tolerant to both TAN and high‐concentration VFA, synergistically promoting hydrolysis and acidogenesis (Figure S8a, Supporting Information). In contrast, the lower abundance of these key genera, unranked MBA03 (3.93%) and unranked Firmicutes (1.32%), in CKG explains its weaker hydrolysis and acidogenesis performance compared to TD1G. These two genera have a crucial role in the hydrolytic and acidogenic process by constructing a microbial symbiotic network (Figure 2b).

Archaeal communities were also driven by rapid hydrolysis and VFA accumulation (Figure S7c, Supporting Information), and the key genera have been identified (Figure 2c). The archaeal community in TIG is primarily composed of *Methanosarcina* ^[^ ^28^ ^]^ (59.98%) and *Methanoculleus* ^[^ ^29^ ^]^ (5.70%), but their methanogenic potential was limited. After inoculating with TAN‐tolerant inoculum, the dominant genera *Methanosarcina* in TD0G gradually increased to 60.97% and coexisted with *Methanoculleus* (5.61%), making their archaeal community similar to that of CKG, which was composed of *Methanosarcina* (64.18%) and *Methanoculleus* (4.82%). However, the difference in CH_4_ production between TD0G and CKG suggests that the methanogenic methanogenesis pathway may be limited by gene expression levels, rather than solely by the abundance of key archaea *Methanosarcina* (Figure S8b, Supporting Information). As the proportion of RMA increases, *Methanosarcina* in TD1G remains dominant, accounting for up to 59.84%. Nevertheless, the increase in *Methanoculleus* abundance confirms the possibility of a shift in methanogenesis, with the hydrogenotrophic methanogenic methanogenesis pathway contributing increasingly to CH_4_ production. The relationship between archaea and bacteria was further analyzed in Figure 2d, *Methanosarcina* and *Methanoculleus* can be co‐enriched with an unranked MBA03 and unranked Firmicutes to produce CH_4_ through symbiotic metabolic network, which is the main metabolic pattern in TD1G.

Therefore, the combination of hydrolysis pretreatment and TAN‐tolerant inoculum facilitates the reconstruction of an efficient microbial community dominated by unranked MBA03, unranked Firmicutes and *Methanoculleus*, achieving CH_4_ production through synergistical hydrolysis‐acidogenesis‐methanogenesis within a symbiotic metabolic network.

### 2.3 Enhancement of Synergistical Hydrolysis‐Acidogenesis‐Methanogenesis

**Figure 3:**
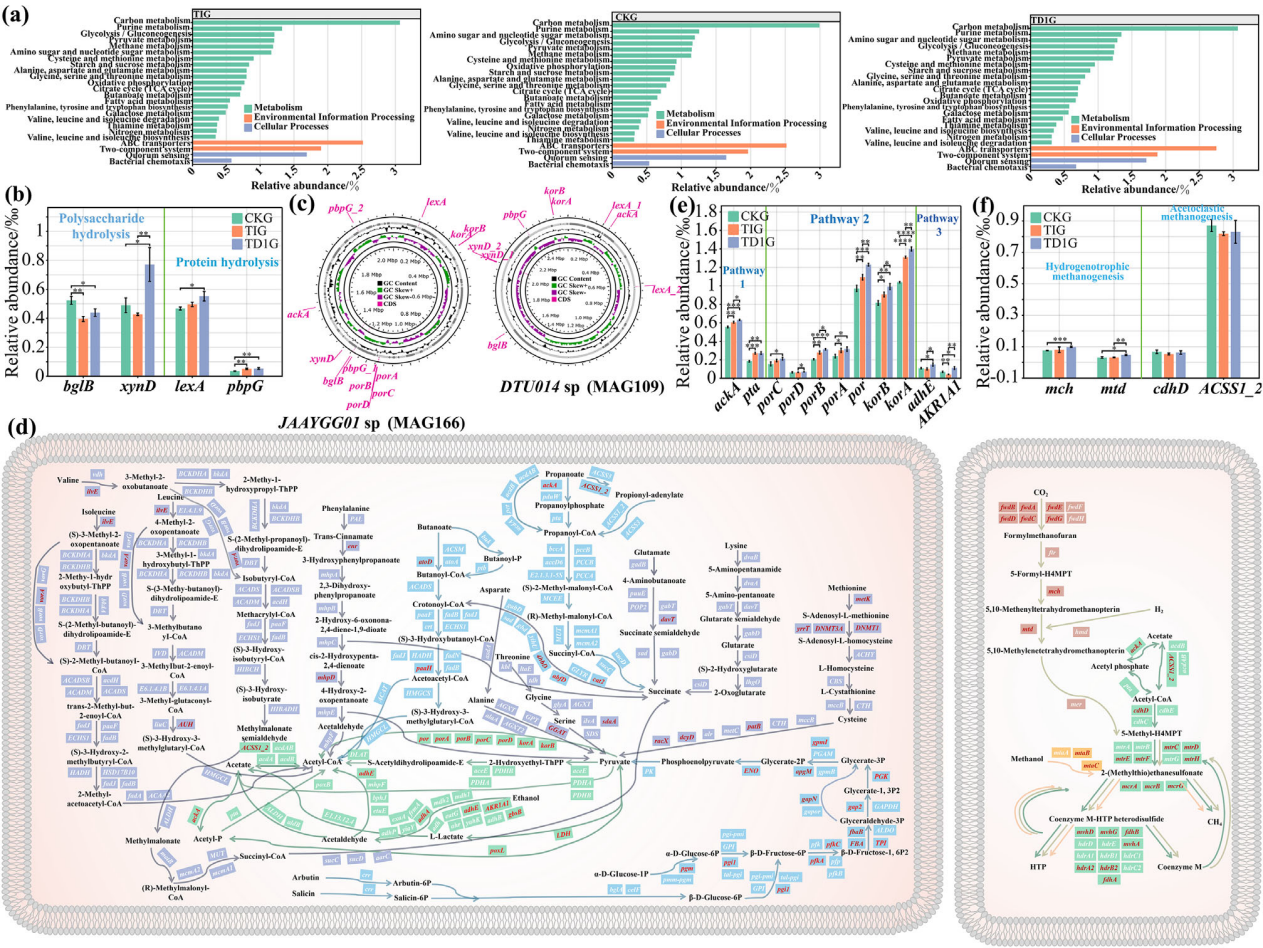
a) Enrichment abundance of metabolic pathways in different AD systems. b) Key genes involved in protein hydrolysis and polysaccharide hydrolysis. c) Circadian diagrams of the genomes of key strains *JAAYGG01* sp. and *DTU014* sp., and the key hydrolytic genes. d) The metabolic pathway diagram involving VFA and CH_4_ production reconstructed based on KEGG. The genes marked by red indicate that the gene is higher than TIG. Dark blue represents amino acid metabolism, light blue represents VFA metabolism, and green represents polysaccharide metabolism. The abundance of key genes involved in e) VFA production and f) CH_4_ production. Significance testing was performed by *t‐*test: ****P < 0.0001; ***P < 0.001; **P < 0.01; and *P < 0.05.

Further metagenomic analysis revealed the vital pathways and genes related to acidification and CH_4_ production in TD1G. Its glycolysis/gluconeogenesis, methane metabolism, and pyruvate metabolism abundance were higher than that in TIG and CKG (**Figure** **3**a), explaining why its AD performance was improved.

The genomic circular map of the key strain belong to phylum Firmicutes (Table S3, Supporting Information), featuring essential genes, was constructed based on metagenome‐assembled genomes. For hydrolysis, the high abundance of unranked Firmicutes (mainly *JAAYGG01* sp. and *DTU014* sp.) led to the significant enrichment of proteolytic genes (*pbpG* and *lexA*) (*P* < 0.05, Figure 3b,c), and the polysaccharide hydrolytic genes (*bglS* and *xynD*) (*P* < 0.05, Figure 3b,c) responsible for lignocellulose (limiting components) degradation. These genes were identified as key hydrolytic genes.

For VFA production, the vital pathways have been reconstructed based on Kyoto Encyclopedia of Genes and Genomes (KEGG) (Figure 3d), along with their abundance of enrichment (Figure S9a,b, Supporting Information). The pathway abundance of methionine metabolized to pyruvate in TD1G (3.07%) exceeded the abundance of TIG (2.93%) and CKG (2.96%), which was considered the main VFA production pathway. Its glycolysis process also achieved enrichment beyond TIG (9.85%) and CKG (9.77%), with an abundance of 9.94%. Importantly, the high abundance of unranked Firmicutes genera (mainly *DTU015* sp., *DTU013* sp., and *JAAYYLO01* sp.) leads to the enrichment of genes in three key steps (Table S3 and Figure S10a–d, Supporting Information). For acetate production, the abundance of genes *pta* and *ackA* in TD1G (0.90%) significantly exceeded (*P* < 0.05, Figure 3e) TIG (0.88%) and CKG (0.74%). For acetyl‐CoA production, the abundances of key genes *porC*, *porD*, *porB*, *porA*, *por*, *korB*, and *korA* carried by the three strains in TD1G were significantly higher than the other two AD groups (*P* < 0.05, Figure 3e). The enrichment of key genes *AKR1A1* and *adhE* strengthens the pathway of ethanol‐acetyl‐CoA‐CH_4_ (*P* < 0.05, Figure 3e).

For CH_4_ production, key pathways were carefully identified (Figure 3d). Key gene loci on the genomic circular map of *Methanoculleus* and *Methanosarcina* have been highlighted (Table S3 and Figure S11a–c, Supporting Information). Due to the high abundance of an unclassified species in the *Methanoculleus* and *Methanosarcina mazei*, the key genes *mch* and *mtd* regulating the hydrogenotrophic methanogenesis were significantly enriched (*P* < 0.05, Figure 3f), resulting in a pathway abundance of 4.55%, surpassing TIG (3.90%) and CKG (3.42%). The enriched acetoclastic pathway assists in CH_4_ production.

In conclusion, under extreme TAN inhibition, the reconstruction of synergistic bacterial and archaeal communities leads to enhanced hydrolysis‐acidogenesis‐methanogenesis synergism, resulting in higher CH_4_ production in TD1G compared to TIG and CKG.

### 2.4 Effective Expression of Key Genes Involved in Synergistical Hydrolysis‐Acidogenesis‐Methanogenesis

**Figure 4:**
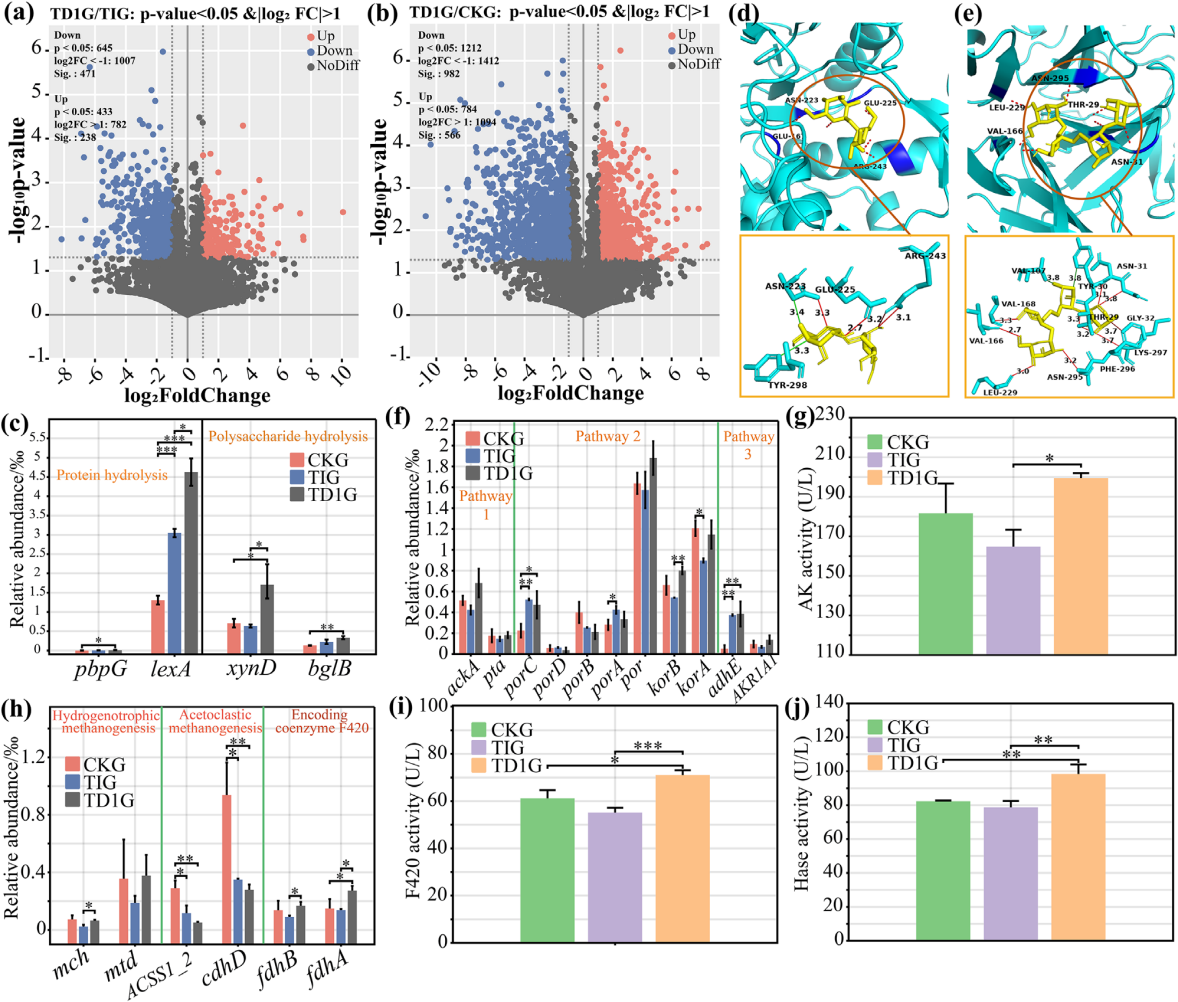
Gene expression volcano map of a) TD1G versus TIG and b) TD1G versus CKG. The red dots indicate the genes that are significantly upregulated, the blue dots indicate the genes that are significantly downregulated, and the gray dots indicate the genes that are not significantly changes. c) Key genes involved in protein hydrolysis and polysaccharide hydrolysis. d) Optimal molecular docking model of cellulose and *bglB*, as well as a schematic diagram of the interaction force. e) Optimal molecular docking model of hemicellulose and *xynD*, as well as a schematic diagram of the interaction force. Enzyme protein, substrate, amino acid residue, hydrogen bond, and hydrophobic force are labeled as cyan, yellow, blue, red, and green, respectively. f) Expression abundance of key genes involved in VFA production. g) AK activity. h) Expression abundance of key genes involved in CH_4_ production. i) F420 activity. j) Hase activity. Significance testing was performed by *t‐*test: ****P < 0.0001; ***P < 0.001; **P < 0.01; and *P < 0.05.

Transcriptome data can confirm whether the pathways of TD1G are effectively expressed. Its pyruvate metabolism, glycolysis/gluconeogenesis and butanoate metabolism were all upregulated compared with TIG and CKG (log_2_FC > 0, *P* < 0.05, Figure S12a,b, Supporting Information). In addition, its upregulated gene number was higher than TIG and CKG with 238 and 566 (log_2_FC > 1.0, *P* < 0.05, **Figure** **4**a,b), respectively, alleviating the inhibition of the whole AD process.

The genes involved in polysaccharide hydrolysis (*xynD* and *bglB*) and proteolysis (*pbpG* and *lexA*) were significantly expressed in TD1G (log_2_FC > 0, *P* < 0.05, Figure 4c), which was further confirmed as key genes. For the hydrolysis mechanism of limiting substrates (lignocellulose), the beta‐glucan endohydrolase (encoded by *bglS*) and arabinofuranosidase (encoded by *xynD*) were used for molecular docking of cellulose and hemicellulose, respectively, and the optimal binding models with binding energies of −6.4 and −8.7 kcal mol^−1^ (Tables S4 and S5, Supporting Information) were obtained. The two hydrolases can stably degrade cellulose (Figure 4d) and hemicellulose (Figure 4e) through hydrogen bonding and hydrophobic interactions.

The VFA and CH_4_ production map was reconstructed based on transcription data with their expressed abundance (Figure S13a,b, Supporting Information). The glutamate metabolism, methionine metabolism, and glycolysis expression levels in TD1G were higher than those in CKG. For the key step of acetate production, the genes *ackA* and *pta* carried by *DTU015* sp., *DTU013* sp., and *JAAYYLO01* sp. in TD1G (0.86‰) were higher than that in TIG (0.57‰) and CKG (0.69‰) (Table S3 and Figure S10a–d, Supporting Information; Figure 4f). The activity of acetate kinase (AK) encoded by vital gene *ackA* reached 199.46 U L^−1^ (Figure 4g), which was also higher than other AD systems. For the key steps of acetyl‐CoA from pyruvate, high abundance of carried by the three strains resulted in *porC*, *porD*, *porB*, *porA*, *por*, *korB*, and *korA* higher enrichment in TD1G (Table S3 and Figure S10a–d, Supporting Information; Figure 4f). The pathway of ethanol metabolized to acetyl‐CoA regulated by *AKR1A1* and *adhE* was higher expressed in TD1G (log_2_FC > 0, Figure 4f), assisting VFA production.

For CH_4_ production, the expression levels of key genes (*mch*, *mtd*) that restrict the hydrogenotrophic methanogenesis pathway in TD1G were more than twice that of TIG, similar to those in CKG (*P* < 0.05, Figure 4h). However, the expression levels of key genes (*ACSS1_2*, *cdhD*) that restrict the acetoclastic methanogenesis pathway in TD1G are only 60% of those in TIG, significantly lower than those in CKG (*P* < 0.05, Figure 4h). This further supports the conclusion from Figure 1 that the hydrogenotrophic methanogenesis pathway contributes to about 93% of CH_4_ production. Importantly, due to key archaea (Table S3 and Figure S11a–c, Supporting Information), *fdhA* and *fdhB* were significantly enriched in TD1G (*P* < 0.05, Figure 4h), with the activity of the coenzyme F420 (F420) they encode for regulating crucial stages of CH_4_ production being significantly higher compared to other AD systems (*P* < 0.05, Figure 4i). The *hydC* was expressed effectively (Figure S14, Supporting Information), and the activity of the [FeFe] hydrogenase (Hase) it encodes for, which regulates H_2_ production, was higher (*P* < 0.05, Figure 4j), assisting in the enhancement of the hydrogenotrophic methanogenesis.

Therefore, the reconstruction of a high enzyme activity microbial community has enriched key hydrolytic genes, subsequently achieving VFA production through glycolysis, pyruvate metabolism and butyrate metabolism, ultimately stabilizing CH_4_ production via the hydrogenotrophic methanogenesis.

### 2.5 Efficient Mass Transfer and Self‐Regulation Assisting Extreme Ammonia Inhibition

**Figure 5:**
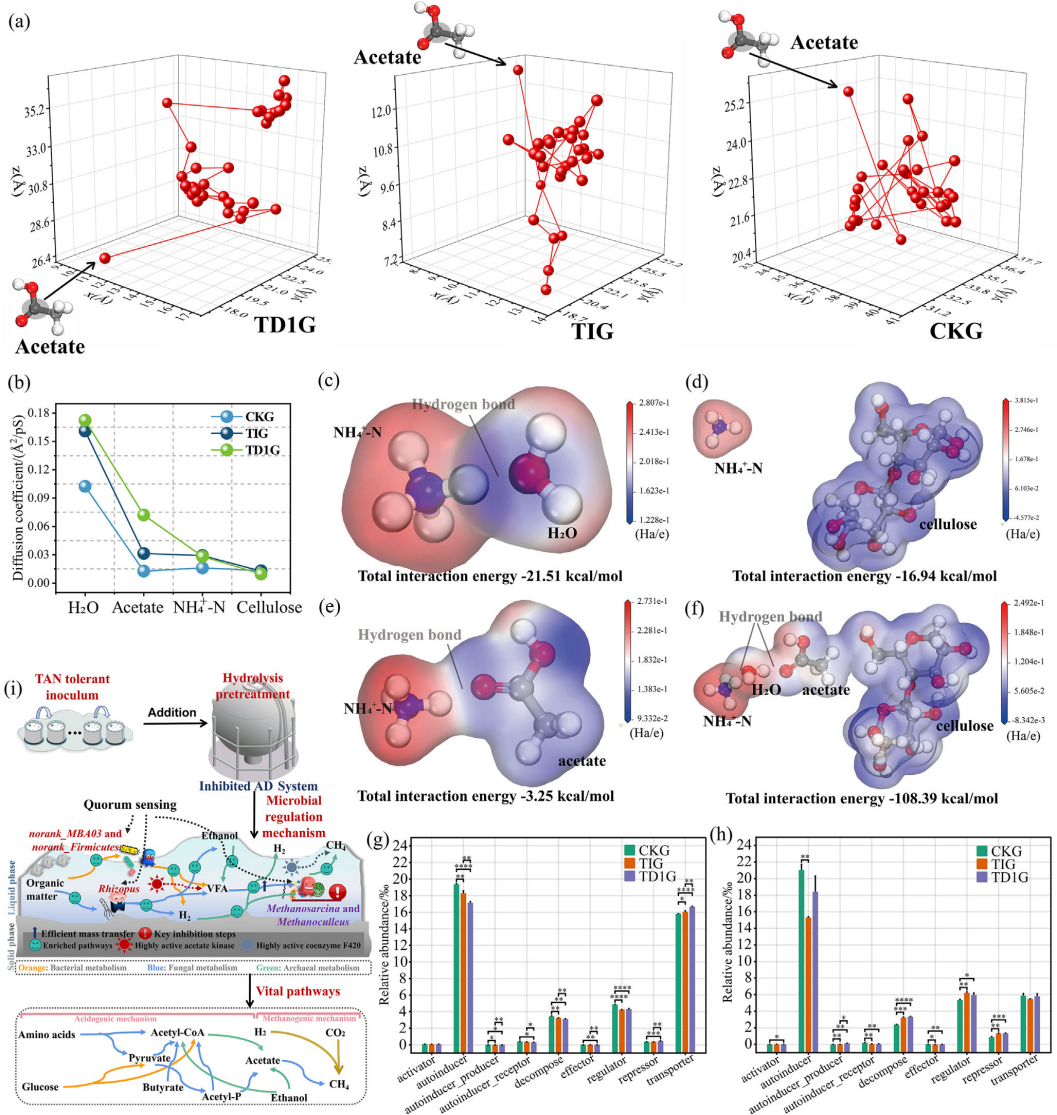
a) Trajectory of carbon atom in a selected acetate molecule in different AD systems. b) Diffusion coefficient of key intermediates in various AD systems. Electrostatic potential penetration and total interaction energy between c) NH_4_^+^‐N molecules and water molecules, d) NH_4_^+^‐N molecules and individual cellulose molecules, e) NH_4_^+^‐N molecules and acetate molecules, and f) a collective consideration of these four types of molecules. g) Abundance of QS function and h) their expression abundance. i) Mechanism of hydrolysis pretreatment coupled with TAN‐tolerant inoculate relieving inhibition of ultrahigh concentration TAN. Significance testing was performed by *t‐*test: ****P < 0.0001; ***P < 0.001; **P < 0.01; and *P < 0.05.

The linear discriminant analysis effect size (LEfSe) was used to reveal why the microbial community of TD1G resists TAN inhibition. The significant difference between TD1G versus TIG, and TD1G versus CKG, which can be explained by the enrichment of ABC transporters with high linear regression analysis (LDA) scores (Figure S15a,b, Supporting Information). For mutual validation, an amorphous cell was constructed based on the experimentally concentration characteristics, and the kinetic features of four types of molecules in the AD system were calculated (Figure S16, Supporting Information). Acetate, as a key intermediate product, had its trajectory visualized (**Figure** **5**a), which revealed a larger diffusion range in TD1G. Further calculation confirms that the diffusion coefficient of acetate molecules in TD1G was higher than that in CKG and TIG, synergizing with the highly expressed microbial transport functions and collectively proven to promote the methanogenesis process (Figure 5b). The lower diffusion coefficient of NH_4_^+^‐N molecules in TD1G compared to TIG is one of the factors contributing to its higher microbial activity (Figure 5b). In addition, the electrostatic potential penetration between NH_4_^+^‐N and key product molecules was visualized, along with their total interaction energy. NH_4_^+^‐N primarily inhibits microbial activity through binding with water molecules and subsequent contact with microorganisms, followed by binding with hydrolyzed cellulose molecules and acetate molecules (Figure 5c–f). This further confirmed that, in the hydrolyzed AD system, the acetoclastic methanogenesis is a critical step limited by TAN.

Importantly, the functional composition of quorum sensing (QS) can regulate the enrichment of these functional pathways and has already been operational in TD1G. The abundance of effector^[^ ^30^ ^]^ and autoinducer^[^ ^31^ ^]^ of QS in TIG inhibited by TAN was lower than that in CKG, resulting in a decreased regulatory activity of QS (Figure 5g). However, its regulatory abundance was higher than TIG, indicating that the microbial community to reduce TAN inhibition through self‐regulation.^[^ ^32^, ^33^ ^]^ The functional expression characteristics of QS revealed by metatranscriptomic sequencing also confirmed enhancement of regulatory performance in TD1G (Figure 5h).

Briefly, it can be concluded that hydrolysis pretreatment coupled with TAN‐tolerant inocula drives the establishment of an efficient bacterial community dominated by *DTU015* sp., *DTU013* sp., *JAAYLO01* sp., *JAAYGG01* sp., and *DTU014* sp. and a stable archaeal community dominated by *Methanosarcina mazei* and an unclassified *Methanoculleus*. The enrichment of these genera led to the accumulation of key hydrolytic genes (*xynD*, *bglB*, *pbpG*, and *lexA*) and crucial acidogenic pathways (glycolysis, pyruvate metabolism, and butyrate metabolism), ultimately establishing an AD system dominated by the hydrogenotrophic methanogenesis for CH_4_ production (Figure 5i). The microbial self‐regulation reduced the sensitivity of the microorganisms to TAN inhibition, ensuring their activity. Also, the efficient material transfer process promotes CH_4_ production.

### 2.6 Application Implications

Microorganism‐based biotechnology always carries the risk of microbial pathogens. RMA addition led to an increase in the abundance of probiotics *Clostridium* sp.,^[^ ^34^ ^]^ along with the decrease of pathogenic bacteria *Pseudomonas aeruginosa* ^[^ ^35^ ^]^ and virulence factors (VFs) (Figure S17a–c, Supporting Information). To verify the risk of disease, factor score analysis was used to evaluate the samples based on VFs abundance. Pre‐analysis confirmed that these data could be used for factor score analysis, and all VFs were classified as pathogenic factors (Tables S6 and S7, Supporting Information). In comparison to TIG, TD1G exhibited a lower contribution weight to this pathogenic factor, resulting in a decrease in pathogenic potential (Table S8, Supporting Information) and revealing a VFs contribution weight rank (Table S9, Supporting Information).

The carbon residue rate of TD1G (60.56%) was lower than that of TIG (76.68%) and CKG (64.45%) (Figure S18a, Supporting Information). According to the calculation of energy budget (as heat)^[^ ^36^ ^]^ (Figure S18b, Supporting Information), the net energy obtained by TD1G was 26.00% higher than that of CKG, while TIG experienced a loss. As a key factor in measuring the application potential of the method, the economic benefits were calculated based on the literature^[^ ^37^ ^]^ (Figure S18c, Supporting Information). By converting thermal energy to electrical energy with an efficiency of 0.42 (at a retail electricity price of US$0.18 kWh^−1^), a highest profit of US$149.02 was ultimately achieved in TD1G conditions than TIG (−US$84.47) and CKG (−US$42.74). The cradle‐to‐gate life cycle assessment confirmed that every 1 m^3^ CH_4_ produced by CKG would significantly increase terrestrial ecotoxicity, human non‐carcinogenic toxicity, global warming, marine ecotoxicity, and freshwater ecotoxicity, but TD1G could reduce these negative impacts by 80% (Figure S19, Supporting Information). Upon evaluation, it was found that during the AD process, electricity consumption contributed the greatest negative impact on the environment (Figures S20 and S21, Supporting Information). However, TD1G could rapidly accumulate CH_4_ and shorten the AD cycle, thereby reducing electrical output. Therefore, TD1G was confirmed more cleaner than TIG and CKG.

**Figure 6:**
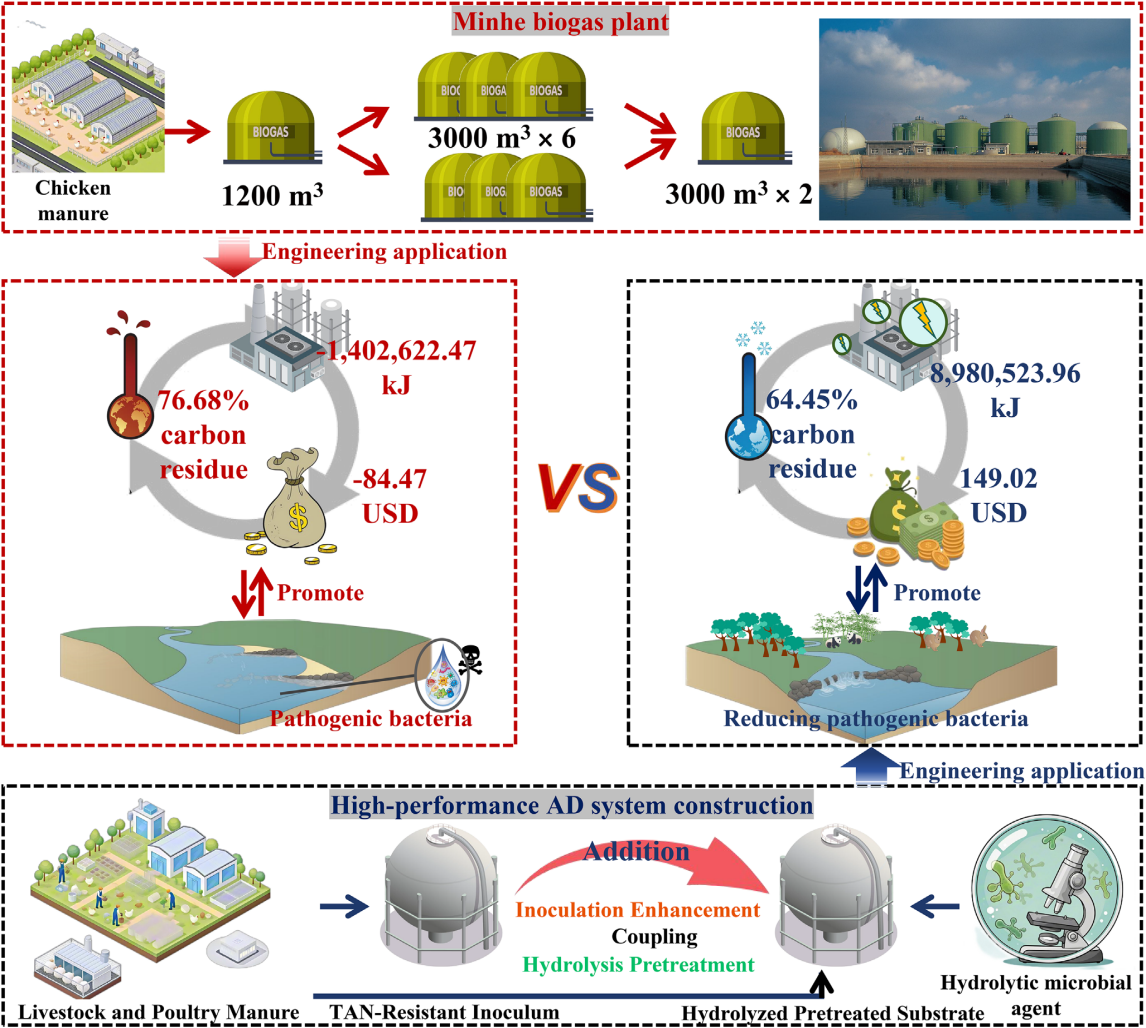
Economic and environmental benefits of coupled strategy enhanced AD compared to Minhe biogas plant.

Further assessment of the application potential of this work was undertaken for a 24 000 m^3^ biogas plant resistant to TAN inhibition in CH_4_ production (Table S10, Supporting Information). In comparison, this work had the potential to increase the power generation per unit TS by 154.64 times. This underscores this work can enhance CH_4_ production and energy conversion efficiency, thereby boosting overall plant operational effectiveness. From the conceptual diagram given in **Figure** **6**, it can be observed that hydrolysis pretreatment coupled with TAN‐tolerant inocula to enhance AD not only reduces pollutants, but also effectively reduces the spread of pathogens. Additionally, it improves carbon utilization, production capacity and economic benefits, providing a safer and more efficient novel strategy.

### 2.7 Machine Learning Optimized Anaerobic Digestion Application

**Figure 7:**
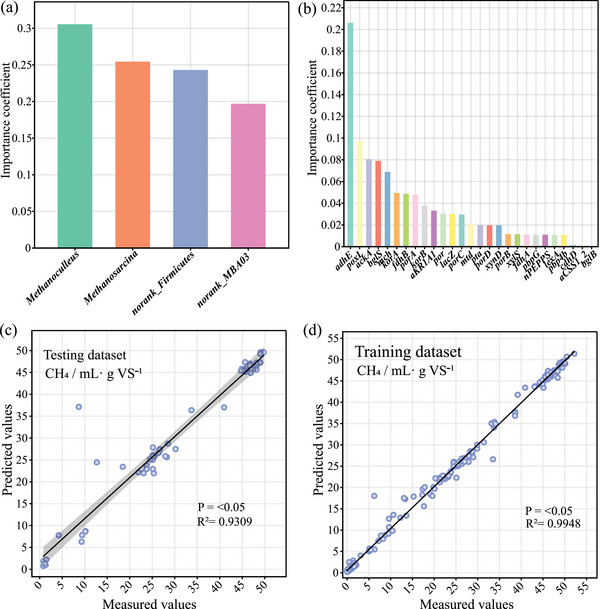
a) Random forest ranking based on general data, b) random forest ranking based on gene data, c) machine learning prediction results based on the test dataset, and d) machine learning prediction results based on the training dataset.

To calculate the priority of factors influencing CH_4_ production, random forest ranking was performed based on microbial taxa and gene data. *Methanoculleus* is crucial for CH_4_ production, confirming that the hydrogenotrophic methanogenesis contributes the most (**Figure** **7**a). In addition, the acetoclastic methanogenesis driven by an unranked Firmicutes, which carries key acetogenic genes, achieves secondary contribution. However, the importance of the enrichment of acidogenic and hydrolytic genes is even more crucial for CH_4_ production than methanogenic genes (Figure 7b). To improve the application efficiency of this strategy, a random forest model was used for machine learning, allowing for the prediction of CH_4_ production based on input parameters like VFA concentration, H_2_ production, and COD concentration, ultimately achieving a fitting accuracy of average *R* ^2^ with 0.96, mean squared error with 12.89, and root mean square error with 3.30 (Figure 7c,d). Attention should be paid to monitoring VFA concentrations to prevent excessive accumulation, thereby reducing CH_4_ production (Figure S22a,b, Supporting Information). This confirmed that machine learning can reflect the complex relationships among concentrated parameters, providing technical guidance for adjusting and designing efficient application strategies.

## 3 Discussion

Ultrahigh‐concentration TAN limits the energy conversion of livestock manure through AD. Although conventional biotechnology can alleviate TAN inhibition, its application remains constrained by its limited efficacy. Methanogenesis is a critical stage inhibited by TAN, directly affecting CH_4_ production. However, merely strengthening the methanogenesis stage cannot completely eliminate the negative impact of TAN, and CH_4_ production cannot be restored to the level when it is not inhibited. It should be noted that the hydrolysis and acidogenesis stages, as the initial steps with CH_4_ production improvement potential, have received little attention. Therefore, by coupling the TAN‐resistant inoculum rich in methanogens with RMA, the entire AD process can be synergistically strengthened, ultimately eliminating TAN inhibition. This study has successfully overcome two significant technical bottlenecks. First, it has developed an innovative strategy sufficient to enhance the entire AD process. Second, it has completely eliminated TAN inhibition through clean biotechnology.

In this work, after hydrolysis‐acidogenesis‐methanogenesis process enhancement, the highest CH_4_ production (196.71 mL/gVS) was obtained. This strategy drives the synergistic reconstruction of microbial communities with high activity dominated by the hydrolytic bacteria *JAAYGG01* sp. and *DTU014* sp., acidogenic bacteria *DTU015* sp., *DTU013* sp., and *JAAYLO01* sp., and the methanogenic archaea *Methanosarcina mazei* and an unclassified species in the *Methanoculleus*. This community efficiently degraded the substrate by enriching critical hydrolytic genes (*xynD*, *bglB, pbpG*, and *lexA*), followed by acetate production regulation with high AK activity through glycolysis, pyruvate metabolism and butyrate metabolism pathways, accompanied by H_2_ release. Ultimately, a stable CH_4_ production was obtained through a hydrogenotrophic methanogenesis pathway with high F420 activity, facilitated by efficient mass transfer processes and quorum sensing regulation. This process has been proven to be cleaner than standard AD processes, with a profit of US$149.02 shift from a loss. Compared to a 24 000 m^3^ biogas plant in Shandong Province, China, with an annual power generation of 22 million kWh, this method has the potential to increase the power capacity by 154.64 times. In addition, the machine learning model provides a theoretical basis for the efficient application of this strategy.

To our knowledge, this study is the first to successfully couple two easily applicable biological methods to completely eliminate TAN inhibition. Not only does this overcome the technical defect of poor effectiveness and long cycles of biotechnology, but also offers significant advantages in reducing health hazards and improving economic benefits. It is important to highlight that our work has been demonstrated to be highly suitable for widespread application and holds significant economic value. This is particularly crucial for factories globally engaged in the resource utilization treatment of agricultural waste.

## 4 Experimental Section

### Source of Waste Activated Sludge, Cow Dung, and Rhizopus Mixed Agent

The WAS was taken from the wastewater treatment plant of Yangling District, Shaanxi Province, China. Tap water was added to adjust the TS and VS. The CD and the RMA were obtained from the Animal Science Experiment Station of Northwest A&F University and Angel Yeast Co., Ltd., China, respectively. **Table** **1** shows the TS, VS, total carbon and nitrogen contents of the material used. RMA did not need to be precultured and could be directly added to the fermenters.

**Table 1:** Characteristics of substrate and RMA.

	TS	VS	Total carbon	Total nitrogen
RMA	94.75%	63.16%	41.77%	1.09%
WAS	6.10%	5.30%	30.38%	5.19%
CD	13.65%	7.50%	36.53%	2.43%

### Experimental Procedure

**Figure 8:**
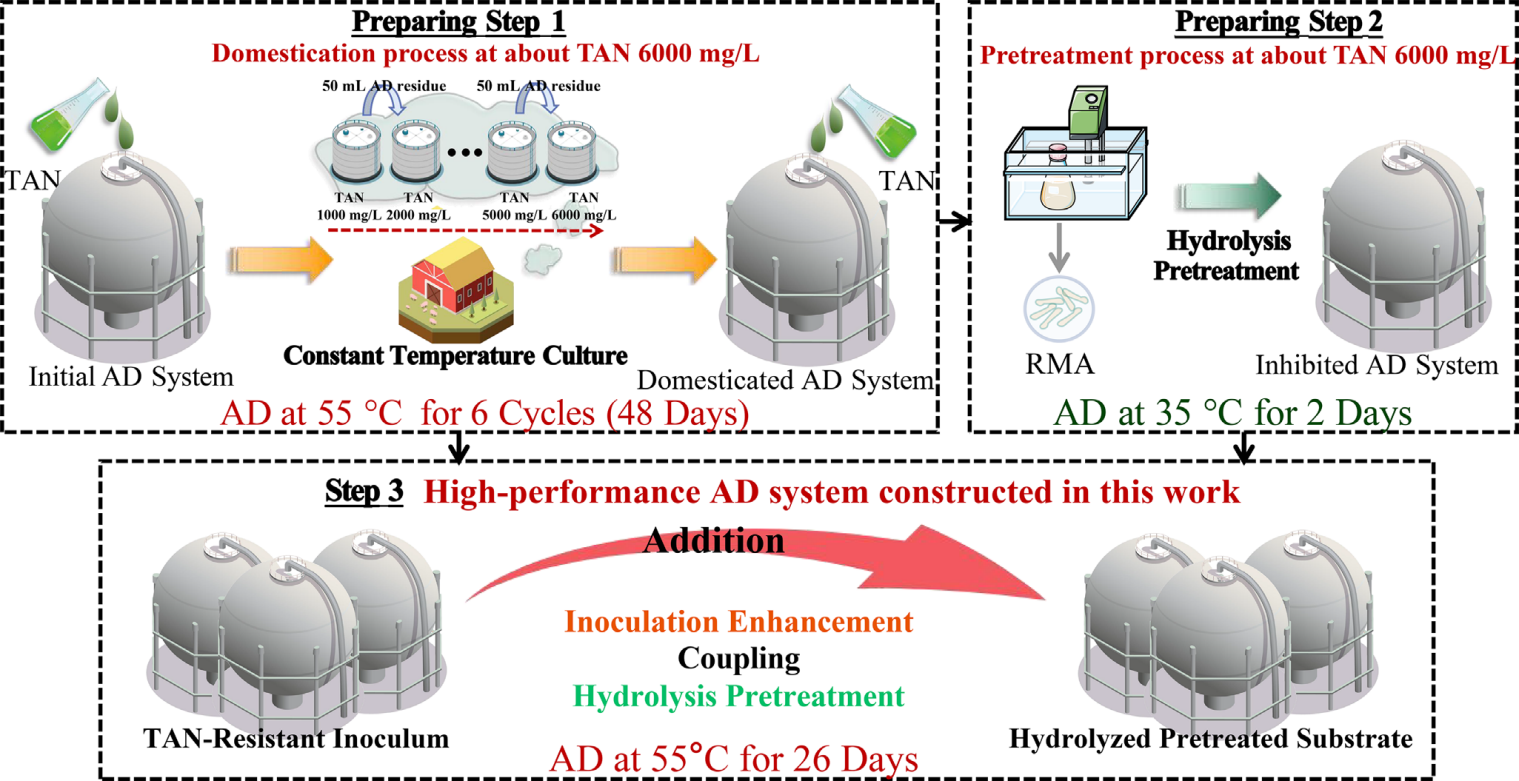
The anaerobic digestion system based on inoculum enhancement coupled with hydrolysis pretreatment, which has completely eliminated total ammonia nitrogen inhibition, was constructed in this work.

As shown in **Figure** **8**, first, a domesticated inoculum with a tolerance to TAN of about 6000 mg L^−1^ cultured under 55 °C was obtained. Second, the substrates in fermenters were hydrolyzed under conditions with RMA at 0, 0.5, 1, 3, and 5 g/g TS for 2 d under 35 °C, respectively. The temperature setting for hydrolysis pretreatment was primarily based on RMA's adaptive range, aiming to fully unleash its hydrolysis potential. Third, the obtained inoculum will be sequentially inoculated into hydrolyzed fermenters with RMA added at 0, 0.5, 1, 3, and 5 g/g TS, and these fermenters will be performed at 55 °C to evaluate CH_4_ production. These fermenters are, respectively, named TD0G, TD0.5G, TD1G, TD3G, and TD5G. To determine the inhibitory effect of TAN on AD, about 6000 mg L^−1^ TAN was added to a fermenter to construct a TAN‐inhibited group, named TIG. A fermenter without TAN was set up to verify whether this work completely eliminates the TAN inhibition, named as CKG. 1‐L fermenters were used for AD within a batch operation mode. The TS of the AD system using WAS and CD is about 6%, and the total volume is 350 mL. Every 2 d, 1 mL of mixed sample was collected from all fermenters and diluted for the determination of NH_4_^+^‐N, COD, CH_4_, H_2_, CO_2_, EC, and VFAs, and the pH of the fermenters was adjusted to about 6.5–7.0. The specific details of the pretreatment experiment are provided in the Supporting Information.

### Verification Experiment

After inactivating, RMA was used as a substrate for AD to verify that the RMA overcomes TAN inhibition by promoting hydrolysis and VFA production rather than acting as a substrate itself. The specific operational details are provided in the Supporting Information. To verify whether acidogenesis and hydrolysis stages were enhanced and to identify the key methanogenic pathways simultaneously, model substrates (glucan, glucose, sodium acetate, H_2_, and methanol) were used for identification, details as follows.^[^ ^38^, ^39^ ^]^ Hydrolysis verification test: a total of 30 mL of the mixture in these fermenters with each RMA inoculation ratio was taken as the inoculum, and then they were diluted ten times. Glucan was used as hydrolytic substrate (5000 mg L^−1^) in the diluted fermenters. TAN, pH, and temperature were consistent with above parameters. CH_4_ production was detected after 2 d. Acidogenic verification test: glucose (5000 mg L^−1^) was used instead of glucan, and the experimental process was consistent with the hydrolysis verification. Acetoclastic methanogenesis test: sodium acetate (5000 mg L^−1^) was used instead of glucan, and the experimental process was consistent with the hydrolysis verification. Hydrogenotrophic methanogenesis test: mixed gas (40% H_2_, 50% N_2_, and 10% CO_2_) was used instead of glucan, and the experimental process was consistent with the hydrolysis verification. The methylotrophic methanogenesis test: methanol (5000 mg L^−1^) was used instead of glucan, and the experimental process was consistent with the hydrolysis verification.

### Assessment of Enzyme Activity in Anaerobic Digestion Systems

The activity of LDH,^[^ ^40^ ^]^ AK,^[^ ^41^ ^]^ Hase,^[^ ^42^ ^]^ and F420^[^ ^43^ ^]^ after AD was measured to represent cell membrane damage, acetate production potential, hydrogenotrophic methanogenesis, H_2_ production potential, and CH_4_ production potential, respectively. All indicators were measured three times.

### DNA Extraction, Polymerase Chain Reaction, and High‐Throughput Sequencing

Total microbial genomic DNA was extracted from samples using the EZNA soil DNA Kit (Omega Bio‐tek, Norcross, GA, USA) according to manufacturer's instructions. The quality and concentration of DNA were determined by 1.0% agarose gel electrophoresis and a NanoDrop2000 spectrophotometer (Thermo Fisher Scientific, Waltham, MA) and kept at −80 °C prior to further use. Polymerase chain reaction (PCR) amplification was carried out using primers 338F (ACTCCTACGGGAGGCAGCAG) and 806R (GGACTACHVGGGTWTCTAAT) for the V3 + V4 region of bacterial 16S rRNA gene. Archaeal DNA was amplified using the forward primer 524F10extF (TGYCAGCCGCCGCGGTAA) and reverse primer Arch958RmodR (YCCGGCGTTGAVTCCAATT). The bacterial PCR reaction mix (20 µL) contained 4 µL of 5× FastPfu Buffer, 2 µL of 2.5 × 10^−3^ m dNTPs, 0.8 µL of forward primer (5 × 10^−6^ m), 0.8 µL of reverse primer (5 × 10^−6^ m), 0.2 µL of BSA, 0.4 µL of FastPfu polymerase, and 10 ng of template DNA. The archaeal PCR reaction mix (20 µL) contained 10 µL of 2×Pro Taq, 0.8 µL of forward primer (5 × 10^−6^ m), 0.8 µL of reverse primer (5 × 10^−6^ m), and 10 ng of template DNA. In the following thermal cycling steps, PCR was performed in ABIGeneAmp 9700 (Applied Biosystems, Carlsbad, CA): initial denaturation at 95 °C for 3 min, then denaturation at 95 °C for 30 s for 27 denaturation cycles, and annealing at 55 °C for 30 s, then extend at 72 °C for 45 s, then at 72 °C for 10 min and 10 °C until the stopped. The PCR product was extracted from 2% agarose gel and purified using the PCR Clean‐Up Kit (YuHua, Shanghai, China) following the manufacturer's instructions and quantified using Qubit 4.0 (Thermo Fisher Scientific). Purified amplicons were pooled in equimolar amounts and paired‐end sequenced on an Illumina PE300/PE250 platform (Illumina, San Diego, CA) according to the standard protocols by Majorbio Bio‐Pharm Technology Co. Ltd. (Shanghai, China).

### Shotgun Sequencing, Genome Assembly, Gene Prediction, Taxonomy, and Functional Annotation

The DNA was fragmented to an average size of 400 bp using Covaris M220 (Gene Company Limited, China) for paired‐end library preparation. The library was constructed with NEXTFLEX Rapid DNA‐Seq (Bioo Scientific, Austin, TX), and paired‐end sequencing was performed on an Illumina NovaSeq at Majorbio Bio‐Pharm Technology Co., Ltd. (Shanghai, China) using the NovaSeq 6000 S4 Reagent Kit v1.5 (300 cycles) following the manufacturer's instructions. All measurements were performed in triplicate. The paired‐end Illumina reads were trimmed of adaptors, and low‐quality reads (length <50 bp, quality value <20, or having N bases) were removed by fastp (https://github.com/OpenGene/fastp, version 0.20.0). Metagenomics data were assembled into contigs (length ≥ 300 bp) using MEGAHIT.^[^ ^44^ ^]^ Open reading frames (ORFs) in each assembled contig were predicted using Prodigal^[^ ^45^ ^]^ or MetaGene^[^ ^46^ ^]^ (http://metagene.cb.k.u‐tokyo.ac.jp). ORFs predicted to be ≥100 bp long were extracted and then translated into amino acid sequences using the NCBI translation table (http://www.ncbi.nlm.nih.gov/Taxonomy/taxonomyhome.html/index.cgi?chapter = tg ncodes#SG1). A non‐redundant gene catalog was compiled with CD‐HIT^[^ ^47^ ^]^ (http://www.bioinformatics.org/cd‐hit, version 4.6.1) with a 90% sequence identity and 90% coverage. SOAPaligner^[^ ^48^ ^]^ was used to compare high‐quality readings with non‐redundant gene catalogs to calculate gene abundance with 95% identity (http://soap.genomics.org.cn, version 2.21). Diamond^[^ ^49^ ^]^ (http://www.diamondsearch.org/index.php, version 0.8.35) was used to compare the representative sequences in the non‐redundant gene catalog with the NR database, and the E‐value cutoff value of 1e^−5^ was used for classification annotation. Diamond^[^ ^49^ ^]^ was used to perform KEGG (http://www.genome.jp/keeg), annotation (http://www.diamondsearch.org/index.php, version 0.8.35), carbohydrate‐active enzymes (CAZy) annotation (http://www.cazy.org), virulence factor annotation (http://www.mgc.ac.cn/VFs), and UniprotKB annotation (http://www.uniprot.org), with an E‐value cutoff value of 1e^−5^. The subsequent binning analysis and taxonomic annotation were conducted using metagenomic data, with detailed methods provided in the Supporting Information.

### Metatranscriptomic Sequencing and Functional Annotation

Sequencing was conducted using the Illumina Novaseq 6000 sequencer (Illumina) and the paired‐end sequencing method from Majorbio Bio‐Pharm Technology Co. (Shanghai, China). The fastp tool^[^ ^50^ ^]^ (version 0.19.6, https://github.com/OpenGene/fastp) was used to trim reads with less than 50 bp and ending with Phred quality scores below 20. SortMeRNA^[^ ^51^ ^]^ (v2.1b, http://bioinfo.lifl.fr/RNA/sortmerna) was used with Trinity^[^ ^52^ ^]^ (version 2.2.0, http://trinityrnaseq.github.io) for de novo assembly and splicing. Transcripts with a length ≥300 bp from each sample were merged to eliminate redundancy, with a CD‐HIT^[^ ^47^ ^]^ identity threshold of 0.95 and a minimum coverage of 0.9. The longest sequence was selected as the representative sequence for each gene. Based on the RSEM‐mapped read counts, the single‐gene abundance for each sample was estimated using RPKM (http://deweylab.biostat.wisc.edu/rsem). Diamond^[^ ^49^ ^]^ (version 0.8.35, http://www.diamondsearch.org/index.php) with an E‐value cutoff of 1e^−5^ was used. Functional annotations (KEGG, CAZy, and UniprotKB) for unigenes were obtained.

### Molecular Docking

The molecular docking steps were as follows.^[^ ^53^ ^]^ According to the shotgun sequencing data and literature,^[^ ^54^ ^]^ the enzyme encoded by *bglS* and *xynD* were selected and their structures were modeled using SWISS‐MODEL (https://swissmodel.expasy.org/interactive) homology modeling. Molecular docking was performed using Auto Dock Tools (https://autodock.scripps.edu). The Auto Dock Tools was used to remove excess solvent molecules and remove any unbound organic contaminants from the selected proteins. The molecular docking of receptors and ligands was performed using Vina software, and the docking results were visualized using Pymol software (https://pymol.org/2).

### Analytical Methods

COD and NH_4_^+^‐N were determined by the potassium dichromate spectrophotometric method, Nessler's reagent spectrophotometric method, respectively. Total carbon and nitrogen were measured by the elemental analyzer (Elementar vario MACRO cube, Germany). The TS and VS were measured by the weight loss method. pH was measured with a pH meter (PHS‐3C, China). The determination of VFAs, H_2_, CO_2_, and CH_4_ used gas chromatograph,^[^ ^55^ ^]^ and the total VFAs was the sum of the measurement value of valerate, isovalerate, butyrate, isobutyrate, propionate, and acetate. The concentration of lactate dehydrogenase and activity of AK, Hase, and F420 were analyzed by assay kits purchased from Shanghai Enzyme‐Linked Biotechnology Co., Ltd. (Shanghai, China) according to the instructions. The concentration of TAN was calculated by S(NH_3_‐N+NH_4_^+^‐N) × 10^pH^/(K_b_/K_w_ + 10 ^pH^),^[^ ^56^ ^]^ where S(NH_3_‐N+NH_4_^+^‐N) is the concentration of NH_3_‐N+NH_4_^+^‐N, *K*_b_ is the ionization constant of the ammonia equilibrium equation and *K*_w_ is the ionization constant of water. The value of *K*_b_/*K*_w_ was calculated via the formula of *K*_b_/*K*_w_ = e^6344/(273 +^ *^T^* ^)^, (*T* = 55 °C during this experiment). EC was measured by a conductivity meter (Youke conductivity DDS‐11A, China). The methods of carbon balance and energy analysis, molecular simulation, machine learning (Figure S23, Supporting Information), and life cycle assessment (Figure S24 and Table S11 Supporting Information) are included in the Supporting Information.

Using Mothur v1.30.1, α‐diversity indices were computed, including observed Chao, Shannon, Shannon evenness, and coverage indices.^[^ ^57^ ^]^ The similarity among the microbial communities in different samples was determined by principal component analysis.^[^ ^58^ ^]^ The redundancy analysis was performed using R package “Vegan” v2.5‐3 package to investigate effect of environmental factors on microbial communities.^[^ ^59^ ^]^ Co‐occurrence networks were constructed to explore interactions among genera.^[^ ^60^ ^]^ LDA based on sample α‐diversity was conducted for species and functional diversity assessment, which can be utilized to evaluate the consistency of species and functional diversity.^[^ ^61^ ^]^ LEfSe (http://huttenhower.sph.harvard.edu/LEfSe) was used to identify significantly enriched functional pathways across different samples.^[^ ^62^ ^]^ Significance testing for the enrichment abundance of bacteria and archaea across different samples was performed with the R package “stats” and Python package “SciPy.”^[^ ^63^ ^]^ Beta‐diversity distance matrices were calculated with Qiime, the R package “stats” and the Python package “SciPy” to reflect the within‐group sample dispersion.^[^ ^64^ ^]^ A volcano plot for genes was generated for data that meet |log_2_FC| > 1.0 and *P* < 0.05 criteria and a pathway volcano plot for data that meet |log_2_FC| > 0 and *P* < 0.05 criteria.

### Statistical Analysis

All samples were assayed in triplicate, and the standard deviation analysis and significance testing (*t*‐test) were conducted using Origin 2021. Significance was evaluated by *t*‐test: ****P < 0.0001, ***P < 0.001, **P < 0.01, and *P < 0.05.

## Conflict of Interest

The authors declare no conflict of interest.

## Author Contributions

H.W.: conceptualization, methodology, formal analysis, investigation, writing – original draft, visualization. H.Z.: formal analysis, visualization. Z.L.: methodology, project administration. T.D.: formal analysis. Y.Y.: resources, conceptualization, supervision, project administration.

## Supporting information



Supporting Information

## Data Availability

The data that support the findings of this study are available on request from the corresponding author. The data are not publicly available due to privacy or ethical restrictions.
