# Supplementary material for: Overcoming Extreme Ammonia Inhibition on Methanogenesis by Artificially Constructing a Synergistically Community with Acidogenic Bacteria and Hydrogenotrophic Archaea
Source: Adv Sci (Weinh). 2025 Mar 31;12(23):2502743. doi: 10.1002/advs.202502743 (PMC12199369; doi:10.1002/advs.202502743)
Supplement: Supplementary file 1 — Supporting Information [file ADVS-12-2502743-s001.docx]

**Overcoming Extreme Ammonia Inhibition on Methanogenesis by Artificially Constructing a Synergistically Community with Acidogenic Bacteria and Hydrogenotrophic Archaea**

**Heng Wu^1^, Huaiwen Zhang^1^, Taili Dong^2^, Zhenyu Li^3^, Xiaohui Guo^1^, Heyu Chen^1^, Yiqing Yao^1^***

*^1^College of Mechanical and Electronic Engineering, Northwest A&F University, Yangling, Shaanxi 712100, PR China*;

*^2^Shandong Min-he Biotechnology Co Ltd, Penglai, 265600, China;*

*^3^Water Technologies Innovation Institute & Research advancement, Saudi Water Authority, 31951, Kingdom of Saudi Arabia.*

**About the author:**

*Corresponding author.

E-mail addresses: [dzhtyao@nwafu.edu.cn](mailto:dzhtyao@nwafu.edu.cn); [dzhtyao@126.com](mailto:dzhtyao@126.com) (Y. Yao)

**Pretreatment experimental method**

***Pretreatment Step One: Method for obtaining TAN-tolerant inoculum.*** In an anaerobic digestion (AD) system with an initial volume of 300 mL and a TS concentration of 6%, the TAN concentration is initially raised to approximately 1000 mg/L in the first stage by adding ammonium chloride. Subsequently, the TAN concentration is increased to 2000, 3000, 4000, 5000, and 6000 mg/L in the second, third, fourth, fifth, and sixth stages, respectively. Each stage lasts for 8 days, totaling 48 days for all six stages. After the completion of the first stage, 50 mL of the remaining material from the AD system is used as an inoculum for the second stage's AD system. This process is repeated, with each subsequent stage utilizing the inoculum from the previous stage. Ultimately, after six stages of gradual domestication, the AD residue that is tolerant to TAN is obtained and can be used to mitigate TAN inhibition on methanogenesis.

***Pretreatment Step Two: Hydrolysis pretreatment method.*** The initial volume of the AD system is 300 mL, with a TS concentration of 6%. Under anaerobic conditions at 35°C, RMA is added to different AD systems at ratios of 0, 0.5, 1, 3, and 5 g/g TS, respectively. The hydrolysis process is then allowed to proceed for two days. The TAN concentration in all fermentation tanks is maintained at approximately 6000 mg/L throughout the process. After hydrolysis pretreatment, rapid and effective hydrolysis can be achieved in the first two days, promoting acidogenesis. Simultaneously, this process drives the construction of microbial communities to achieve long-term hydrolysis and enhanced acidogenesis.

**Verification Experiment Method**

***RMA inactivation to verify the strengthening effect of its activity on AD*.** To verify that the RMA overcomes TAN inhibition by promoting hydrolysis and VFA production rather than acting as a substrate itself, inactivated RMA was inoculated into the AD system to assess whether the CH_4_ production from inactivated RMA exceeded that from non-inactivated RMA, and this was compared with CKG to determine if TAN inhibition was alleviated. Since TD1G exhibited the highest CH_4_ production efficiency, a TD1G-inactivation group was established as a comparison. The inactivation steps for the RMA included high-temperature and high-pressure steam sterilization at 121°C for 30 minutes, followed by drying at 105°C for 48 hours. The remaining experimental procedures were kept consistent with those of TD1G.

**Analysis Method of Carbon Utilization, Capacity Production and Economic Benefits**

***Carbon balance calculation (g)****.* The calculation of carbon balance theory is carried out in a relatively ideal state, without considering the loss of trace carbon caused by manual operation during the experiment. It is believed that the total carbon in the AD system mainly comes from gas (CH_4_, CO_2_), liquid (volatile fatty acid (VFA), ethanol), and residual solid (residual carbon). The mass of carbon in CH_4_ and CO_2_ is calculated by the density at 25°C ideal state.

Carbon balance equation: *m*_CD_ + *m*_WAS_ + *m*_RMA +_*m*_inoculum_ = *m*_CH4_ + *m*_CO2_ + *m*_VFA_ + m_ethanol_ + *m*_residual solid_ + *m*_microbial consumption_ (1)

where: *m*_CD_, *m*_WAS_, *m*_RMA_, *m*_inoculum_ represent the quality of carbon in cow dung (CD), waste activated sludge (WAS), RMA, and inoculum respectively, and their sum represents the initial total carbon quality of AD system (g). The *m*_CH4_, *m*_CO2_, *m*_VFA_, *m*_ethanol_, *m*_residual solid_, and *m*_microbial consumption_ represent the carbon mass used by microorganisms in CH_4_, CO_2_, VFA, ethanol, AD remaining solids, and microbial consumption, respectively (g).

The calculation of carbon mass in solid:

*m*_CD_=TC × *m*_existing_ (2)

where: TC represents the measured total carbon content in CD (%), and m_existing_ represents the mass of CD existing in the experiment (g). According to this formula, *m*_WAS_, *m*_RMA_, *m*_inoculum_ and *m*_residual solid_ can be calculated.

The calculation of carbon mass in gas and liquid:

*m*_CH4_=*V*×*ρ*× *η* (3)

where: *V* represents the volume of CH_4_ (m^3^), *ρ* represents the density of CH_4_ at standard atmospheric pressure (g/m^3^), and *η* represents the relative content of carbon in CH_4_. According to this formula, *m*_CH4_, *m*_CO2_, *m*_VFA_, and m_ethanol_ can be calculated. When calculating the carbon mass in the liquid, only the liquid concentration is needed instead of the gas density.

***Energy balance calculation (in heat, kJ)****.* The energy output mainly comes from the combustion heat of H_2_ and CH_4_. The energy input mainly comes from the electric energy consumed by the incubator. Considering the huge volume of the incubator, in order to evaluate the energy production efficiency of this work as accurately as possible, the actual production of H_2_ and CH_4_ will be expanded by 250 times. The thermal efficiency of H_2_ and CH_4_ is calculated according to 143MJ/m^3^ and 37MJ/m^3^.

Energy balance equation:

*E*_output_-*E*_input_=*E*_production_  (4)

*E*_output_=*E*_H2_+*E*_CH4_  (5)

where: *E*_H2_ and *E*_CH4_ represent the heat energy of H_2_ conversion and CH_4_ conversion, respectively, and the sum of the two is the energy output (*E*_output_) of the AD system (kJ). *E*_input_ is the electrical energy consumed by the incubator. *E*_production_ is net capacity (kJ).

*E*_H2_=*V*×*K*_H2_ (6)

*E*_CH4_=*V*×*K*_CH4_  (7)

where: *V* is the volume of H_2_ or CH_4_ (m^3^), *K*_H2_ and *K*_CH4_ are the energy conversion coefficients of the two, which are 143MJ/m^3^ and 37MJ/m^3^, respectively [1].

***Economic benefit calculation (USD).*** The efficiency of converting thermal energy into electrical energy is calculated according to 0.42 [2]. The cost of RMA was calculated according to 27.5 USD/kg, and the electricity cost was calculated according to 0.18 USD/kW.

*Y*_output_-*Y*_input_=*Y*_production_ (8)

*Y*_output_=*Y*_H2_+*Y*_CH4_ (9)

*Y*_inoutput_=*Y*_Electricity_+*Y*_RMA costs_ (10)

*Y*_Electricity_ and *Y*_RMA_ costs represent the cost of electricity and RMA purchase, respectively, while *Y*_H2_ and *Y*_CH4_ represent the benefits of H_2_ and CH_4_ production capacity, respectively.

*Y*_H2_=0.42×*E*_H2_×0.18 (11)

*Y*_CH4_=0.42×*E*_CH4_×0.18 (12)

*Y*_RMA costs_=*m*_RMA_×2.38 (13)

where: *E*_H2_ and *E*_CH4_ can be calculated based on equations (6)-(7) (USD), and *m*_RMA_ is the mass consumed by RMA (g)*. Y*_Electricity_is about 89.86 (USD).

**Metagenomic Binning and Taxonomic Annotation of Bins**

The binning analysis employed metagenomic sequencing data to identify significant bacterial strains and their key genes. Contigs that were 1000 bp or longer were selected as the final assembly output. These contigs underwent further binning to obtain metagenome-assembled genomes (MAGs) using the MetaBAT tool (version 2.12.1) [3]. The results from this binning process were then integrated with DAS_tools (version 1.1.0) [4] to generate an optimized, non-redundant set of bins, constituting the final binning result. Contamination in each bin was assessed by analyzing the GC content, coverage, and tetranucleotide frequency of the included contigs. Based on these assessments, higher-quality contigs were selected, and contaminated sequences were manually removed to enhance the bins' accuracy. The completeness, contamination, and strain heterogeneity of the bins were evaluated using CheckM [5], with only those bins exhibiting over 50% completeness retained for further analysis. Finally, the taxonomic classification of the recovered genomes was determined using a set of 120 universal single-copy proteins sourced from the Genome Taxonomy Database via GTDB-Tk [6]. A genomic circular diagram for the significant bacterial strains was generated using CGview (https://paulstothard.github.io/cgview/).

**Molecular Simulation Method**

***Amorphous Cell Box Model Construction.*** The simulations were conducted using the Forcite module in Materials Studio 2020 software. The molecules used for constructing the amorphous cell model include NH_4_^+^-N molecules, H_2_O molecules, C₆HO₅ molecules, and acetate molecules. Charges were assigned using the COMPASS III electric field, where conventional molecules have a net charge of zero, and NH_4_^+^-N molecules carry a positive charge. An amorphous cell was constructed based on the average solvent composition during the AD process, and due to computational limitations, the concentration of each component was increased by 40 times. Specifically, TD1G contains 7 acetate molecules, 77 NH_4_^+^-N molecules, 74 C₆HO₅ molecules, and 271 H_2_O molecules; CKG contains 1 acetate molecule, 7 NH_4_^+^-N molecules, 90 C₆HO₅ molecules, and 270 H_2_O molecules; TIG contains 2 acetate molecules, 68 NH_4_^+^-N molecules, 90 C₆HO₅ molecules, and 268 H_2_O molecules. The density was set to 1.04 g/cm³ according to the TS concentration. The amorphous cell dimensions are 38.9Å × 38.9Å × 38.9Å. The simulation was set to output 10 frames.

***Geometric Optimization*, *Molecular Dynamics Simulation and Diffusion Coefficient Calculation.*** The optimal frames from the amorphous cell were selected for geometric optimization. The electric field parameters remained unchanged. The maximum number of iterations was set to 5000, and upon completion, a "normal" status indicated that annealing was not required. The optimized frames were then selected for molecular dynamics simulation. The simulation was conducted under constant volume and temperature (NVT) ensemble with a temperature set at 328.15K. The simulation time step was 1 fs, and the total simulation time was 300 ps, with 10,000 steps output per frame. Based on the results obtained from the molecular dynamics simulation, various small molecular systems within the amorphous cell were selected. The stable period between 10-300 ps was chosen for analyzing the Mean Square Displacement (MSD) to calculate the diffusion coefficients.

***Interaction Energy Calculation and Electrostatic Potential Penetration.*** Interaction energy simulations were performed using the Forcite module in Materials Studio 2020 software. Different small molecule models were constructed, followed by geometric optimization. Subsequently, the interaction energy of the molecular system was calculated.

Electrostatic potential penetration simulations were conducted using the Dmol3 module in Materials Studio 2020 software. Various small molecule models were constructed, followed by geometric optimization. Then, the electron density and electrostatics of the molecular system were calculated. The functional used was GGA-BLYP, and the pseudopotential was set to DFT Semi-core Pseudopots. After the calculation, visualization optimization was performed, and hydrogen bonds were annotated.

**Method for determining Lactate dehydrogenase concentration and key enzyme activity using kits**

***Lactate dehydrogenase (LDH) determination***. This kit employs a double antibody sandwich method to determine the LDH concentration in samples. Purified LDH antibodies are used to coat microplates, creating solid-phase antibodies. LDH is then sequentially added to the microplate wells coated with monoclonal antibodies, followed by binding with horseradish peroxidase-labeled LDH antibodies to form an antibody-antigen-enzyme-labeled antibody complex. After thorough washing, the substrate tetramethylbenzidine is added for color development. Under the catalysis of horseradish peroxidase, tetramethylbenzidine turns blue and subsequently converts to a final yellow color under acidic conditions. The intensity of the color is positively correlated with the LDH concentration in the sample. The absorbance (OD value) is measured at a wavelength of 450 nm using a microplate reader, and the LDH activity concentration in the sample is calculated through a standard curve.

***Acetate kinase activity.*** The measurement steps are identical to those for LDH, with the exception of replacing the LDH antibodies with AK antibodies.

***F420 activity.*** The measurement steps are identical to those for LDH, with the exception of replacing the LDH antibodies with F420 antibodies.

***[FeFe] hydrogenase activity.*** The measurement steps are identical to those for LDH, with the exception of replacing the LDH antibodies with [FeFe] hydrogenase antibodies.

**The method for determining VFA is as follows**: The concentrations of various VFAs including acetate, propionate, butyrate, isobutyrate, valerate, and isovalerate were determined by employing a Shimadzu GC-2014C gas chromatograph (Shimadzu Corporation, Kyoto, Japan). This chromatograph was equipped with a DB-FFAP capillary column sourced from Agilent Technologies (Wilmington, DE, USA) and featured a flame ionization detector. Prior to analysis, samples underwent dilution, pretreatment with metaphosphoric acid, and the addition of crotonic acid as an internal measurement standard. The analytical process began at an initial temperature of 50°C, maintained for 3 minutes, and then gradually increased. First, it rose to 130°C at a 10°C per minute rate, then to 170°C at 5°C per minute, and finally reached 220°C at 15°C per minute, stabilizing at this temperature for an additional 3 minutes. For VFA quantification, an internal standard method was adopted. A standard sample was prepared by mixing acetate, propionate, butyrate, isobutyrate, valerate, and isovalerate, sourced from Maiklin Biotech Co., Ltd. in China, and adding crotonic acid as the internal standard. Following gas chromatography analysis, the specific retention times for each acid were accurately recorded.

**The method for determining gas is as follows**: The key gases present in biogas, namely CH_4_, CO_2_, and H_2_, were quantified using a Shimadzu GC-2014C gas chromatograph (produced by Shimadzu Corporation, Kyoto, Japan) outfitted with a Shimadzu MS-13X packed column. The chromatographic conditions were precisely set with a column temperature maintained at 80°C, a DTCD1 temperature of 150°C, and a DINJ temperature of 100°C. Once the temperature ramp was completed, the biogas sample was analyzed directly, enabling the determination of retention times for the various gas components. For calibration purposes, a synthetic biogas mixture, sourced from Shaanxi Maichi Trading Co., Ltd., served as the standard gas.

**

**

**Figure S1**. Visualization of significant tests for NH_4_^+^-N concentration (a), fluctuation in free ammonia nitrogen (FAN) concentration (b), visualization of significant tests for FAN concentration (c), visualization of significant tests for COD concentration (d), fluctuation in EC values (e), visualization of significant tests for EC values (f), visualization of significant tests for VFA concentration (g), fluctuation characteristics of different VFA components (h-n), fluctuation in ethanol concentration (o), visualization of significant tests for ethanol concentration (p), visualization of significant tests for daily H_2_ production (q), and visualization of significant tests for daily CH_4_ production (r) under different AD conditions. The width of the transparent color ribbon represents the error interval. CK was used for *t*-test analysis with other experimental groups, and marker **a** indicated significant differences (P<0.05).


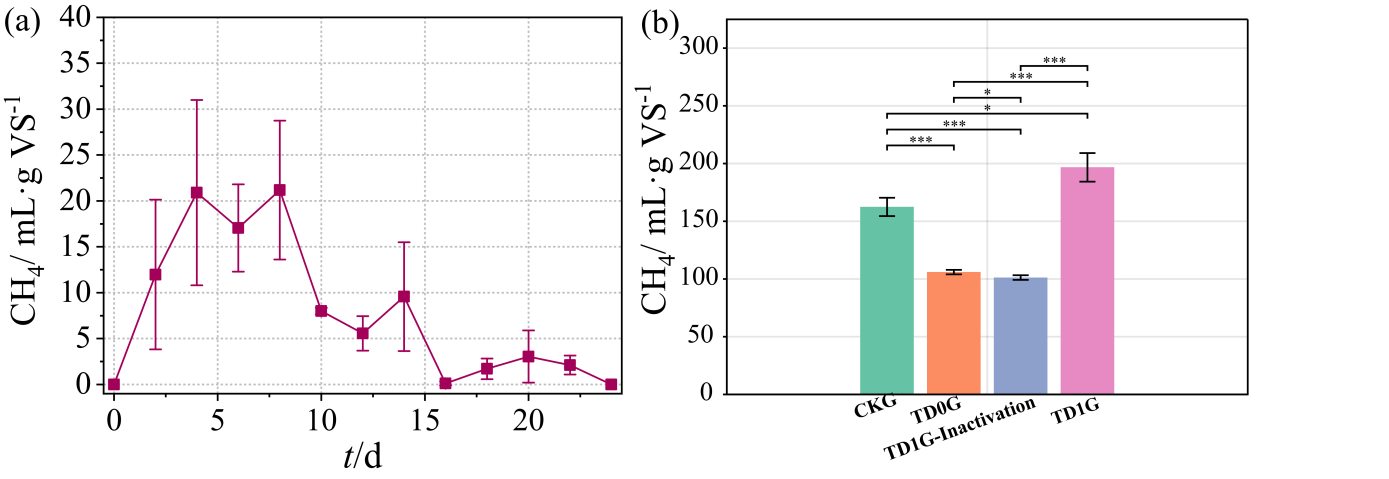


**Figure S2**. The daily CH_4_ production of TD1G based on inactivated RMA-based mixed microbial preparation (a) and the cumulative CH_4_ production of TD1G based on inactivated RMA-based mixed microbial preparation (b). The significance test was performed by *t* test, * * * * means P<0.0001, * * * means P<0.001, * * means P<0.01, * means P < 0.05.

**
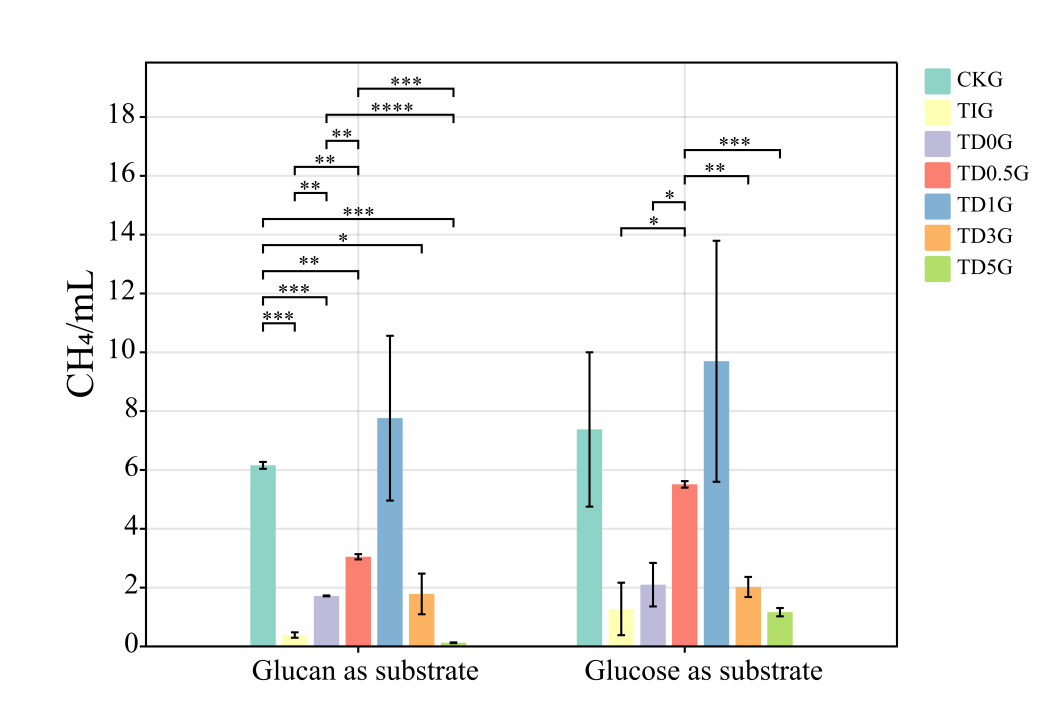
**

**Figure S3**. Verification of hydrolysis (glucan) and acidogenisis (glucose) potential. The significance test was performed by *t* test, * * * * means P<0.0001, * * * means P<0.001, * * means P<0.01, * means P < 0.05.

**Table S1**. Bacterial community alpha diversity under different conditions

|  | chao | shannon | shannoneven | coverage |
| --- | --- | --- | --- | --- |
| CKG | 418.3301267 | 4.223558333 | 0.707405 | 0.998513667 |
| TIG | 394.7693983 | 4.170154 | 0.699333333 | 0.998974 |
| TD0G | 430.1594747 | 4.340667 | 0.721048 | 0.998460667 |
| TD0.5G | 390.392186 | 3.997893 | 0.675268667 | 0.998421333 |
| TD1G | 335.2184947 | 3.953004333 | 0.680686333 | 0.999566 |
| TD3G | 242.919192 | 3.478852 | 0.640910333 | 0.998895 |
| TD5G | 222.4909357 | 3.341596667 | 0.620903 | 0.999394667 |

**Table S2**. Archaeal community alpha diversity under different conditions

|  | chao | shannon | shannoneven | coverage |
| --- | --- | --- | --- | --- |
| CKG | 31.8 | 1.320968667 | 0.385039333 | 0.999872 |
| TIG | 32 | 1.478314 | 0.428526333 | 0.999968 |
| TD0G | 24.91666667 | 1.433553333 | 0.447748333 | 0.999952 |
| TD0.5G | 24.111111 | 1.342411 | 0.421054333 | 0.999936 |
| TD1G | 27 | 1.433797333 | 0.435189333 | 0.999984 |
| TD3G | 20.66666667 | 1.163572667 | 0.384576 | 0.999984 |
| TD5G | 21.33333333 | 1.248224 | 0.407338333 | 0.999984 |

**⁎**The index reflecting community richness is chao. The index that reflects the evenness of the community is shannoneven. The index reflecting community diversity is shannon. The index reflecting community coverage is coverage.

**Table S3.** The species relationship of key microorganisms

| Types | Phylum level | Genus level | Species level |
| --- | --- | --- | --- |
| Fungi | Mucoromycota | *Rhizopus* | *Rhizopus_arrhizus* |
| Fungi | Mucoromycota | *Mucor* | *Mucor ambiguus* |
| Bacteria | Firmicutes | *DTU015* | *DTU015* sp*.* |
| Bacteria | Firmicutes | *DTU013* | *DTU013* sp*.* |
| Bacteria | Firmicutes | *JAAYLO01* | *JAAYLO01* sp. |
| Bacteria | Firmicutes | *JAAYGG01* | *JAAYGG01* sp. |
| Bacteria | Firmicutes | *DTU014* | *DTU014* sp. |
| Archaea | Halobacterota | *Methanosarcina* | *Methanosarcina_mazei* |
| Archaea | Halobacterota | *Methanoculleus* | *unclassified_Methanoculleus* |

**Table S4.** Molecular docking results of cellulose and *bglB*-encoding enzyme

| mode | affinity | dist from best mode | |
| --- | --- | --- | --- |
|  | (kcal/mol) | rmsd l.b. | rmsd u.b. |
| 1 | -6.4 | 0.000 | 0.000 |
| 2 | -6.2 | 2.237 | 3.291 |
| 3 | -6.1 | 2.714 | 4.470 |
| 4 | -6.0 | 1.803 | 6.004 |
| 5 | -5.8 | 2.369 | 6.667 |

**Table S5.** Molecular docking results of lignin and *xynD*-encoding enzyme

| mode | affinity | dist from best mode | |
| --- | --- | --- | --- |
|  | (kcal/mol) | rmsd l.b. | rmsd u.b. |
| 1 | -8.7 | 0.000 | 0.000 |
| 2 | -8.5 | 2.500 | 4.990 |
| 3 | -8.4 | 3.885 | 5.254 |
| 4 | -8.4 | 3.379 | 7.266 |
| 5 | -8.3 | 2.912 | 6.523 |

**Table S6.** KMO ＆ Bartlett test

| KMO value | | 0.584 |
| --- | --- | --- |
| Bartlett sphericity test | Approximate Chi-squared value | 57.915 |
|  | *df* | 3 |
|  | *p* value | 0.000 |

Firstly, it analyzes whether the research data is suitable for factor analysis. It can be seen from the above table that KMO is 0.640, greater than 0.6, which meets the premise requirements of factor analysis, meaning that the data can be used for factor analysis research. And the data passed the Bartlett sphericity test (p < 0.05), indicating that the research data were suitable for factor analysis.

**Table S7.** Variance interpretation rate table

| Factor | Eigenvalue | | | Variance interpretation rate before rotation | | | Variance interpretation rate after rotation | | |
| --- | --- | --- | --- | --- | --- | --- | --- | --- | --- |
|  | Eigenvalue | Variance interpretation rate % | Cumulative % | Eigenvalue | Variance interpretation rate % | Cumulative % | Eigenvalue | Variance interpretation rate % | Cumulative % |
| 1 | 2.812 | 93.719 | 93.719 | 2.812 | 93.719 | 93.719 | 2.812 | 93.719 | 93.719 |
| 2 | 0.171 | 5.685 | 99.405 | - | - | - | - | - | - |
| 3 | 0.018 | 0.595 | 100.000 | - | - | - | - | - | - |

The above Table S7 analyzes the factor extraction and the amount of information extracted by the factor. From the above table, it can be seen that only one factor is extracted by factor analysis. The cumulative variance interpretation rate after rotation is 95.966%. Since the common feature of all functional phenotypes is pathogenicity, this factor is a pathogenic factor.

**Table S8**. Linear combination coefficient and weight results

| Name | Pathogenic factor | Composite score coefficient | Weight |
| --- | --- | --- | --- |
| Eigenvalue ( after rotation ) | 2.812 |  |  |
| Explanation rate of variance | 93.72% |  |  |
| TD1G | 0.5724 | 0.5724 | 33.05% |
| CKG | 0.5667 | 0.5667 | 32.72% |
| TIG | 0.5927 | 0.5927 | 34.22% |

**Table S9.** The composite score of different pathogenic functions

| Composite score | Factor score | Pathogenic bacteria |
| --- | --- | --- |
| 2.23 | 2.23 | LOS (CVF494) |
| 1.70 | 1.70 | Type IV pili (VF0082) |
| 1.07 | 1.07 | FbpABC (VF0272) |
| 0.53 | 0.53 | Beta-hemolysin/cytolysin (CVF171) |
| 0.17 | 0.17 | HitABC (VF0268) |
| 0.17 | 0.17 | Polar flagella (CVF786) |
| 0.18 | 0.18 | Trehalose-recycling ABC transporter (CVF651) |
| -0.62 | -0.62 | Flagella (VF0273) |
| -0.76 | -0.76 | Flagella (VF0430) |
| -0.63 | -0.63 | LPS (VF0542) |
| -0.65 | -0.65 | Alginate (VF0091) |
| -0.88 | -0.88 | MgtBC (VF0106) |
| -0.77 | -0.77 | PhoP/R (CVF331) |
| -0.89 | -0.89 | Flagella (VF0519) |
| -0.84 | -0.84 | HSI-I (VF0334) |

**Table S10**. Operating parameters of Minhe plant

| Process | Temperature | AD substrate | Operation volume | TS | CH_4_ production |
| --- | --- | --- | --- | --- | --- |
| Continuous stirred tank reactor | 37℃ | Chicken manure | 300 t/d | 10% | 310 mL/gVS |

**
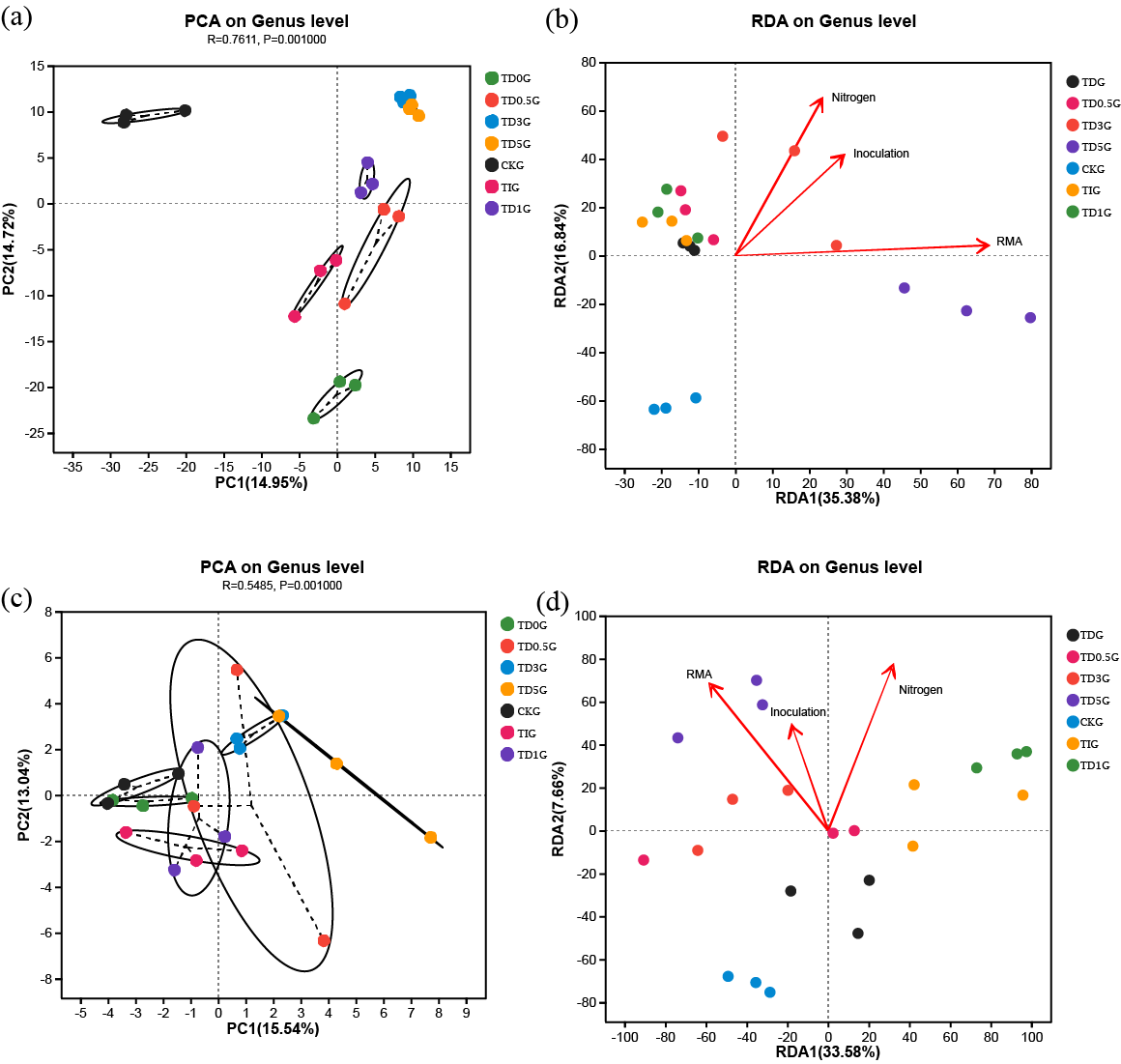
**

**Figure S4**. Principal component analysis (a) and redundancy analysis (b) of bacterial communities, and principal component analysis (c) and redundancy analysis (d) of archaeal communities, under different RMA inoculation conditions. RMA addition rario, whether to add RMA, and TAN concentration respectively represent three influencing factors.


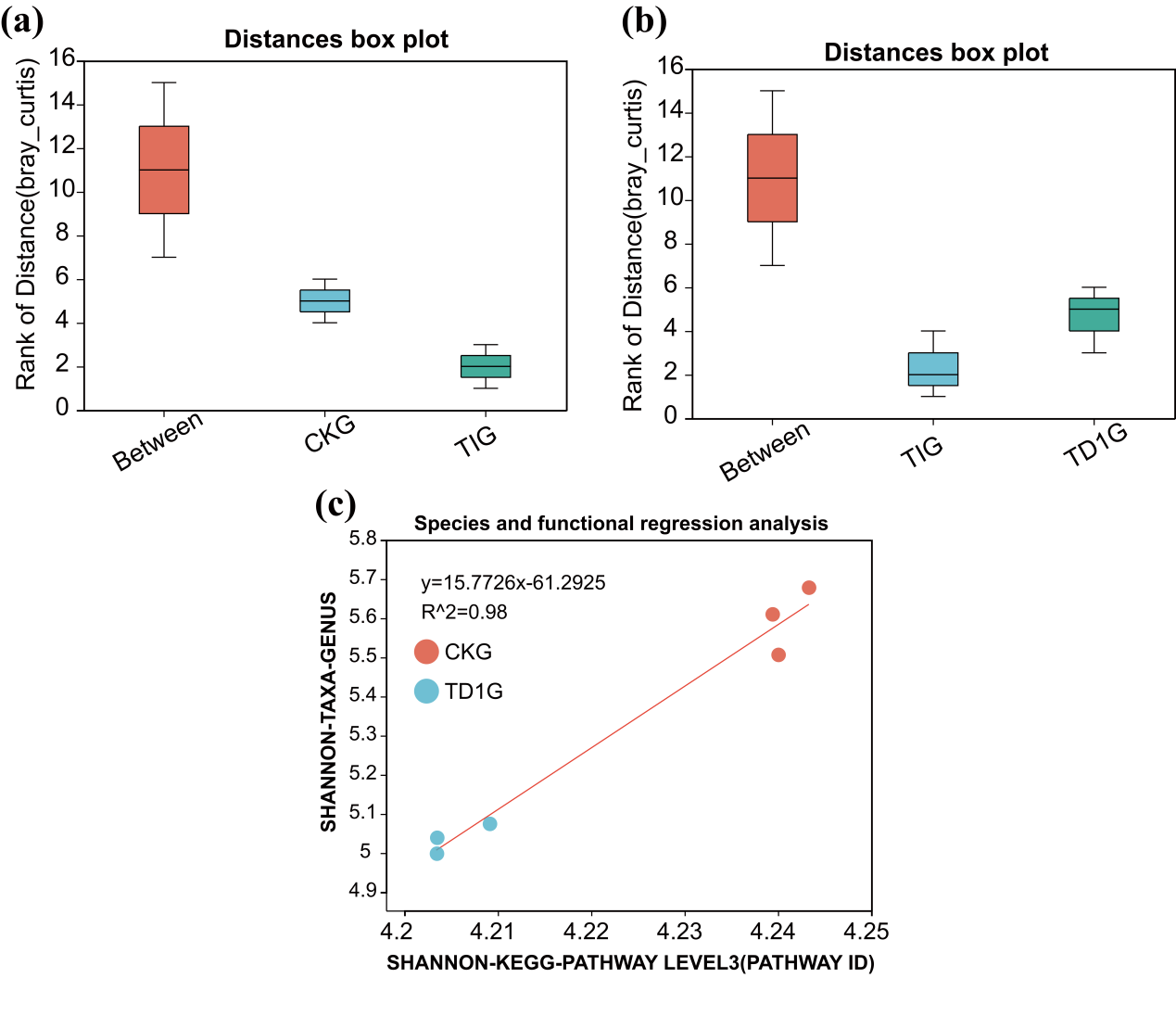


**Figure S5**. The differences between groups and within groups of CKG and TIG samples were shown. Between refers to the difference between groups, and others represent the differences within each group. The ordinate represents the distance value (a). The differences between and within TD1G and TIG samples were shown. Between refers to the difference between groups, and others represent the differences within each group. The ordinate represents the distance value (b).


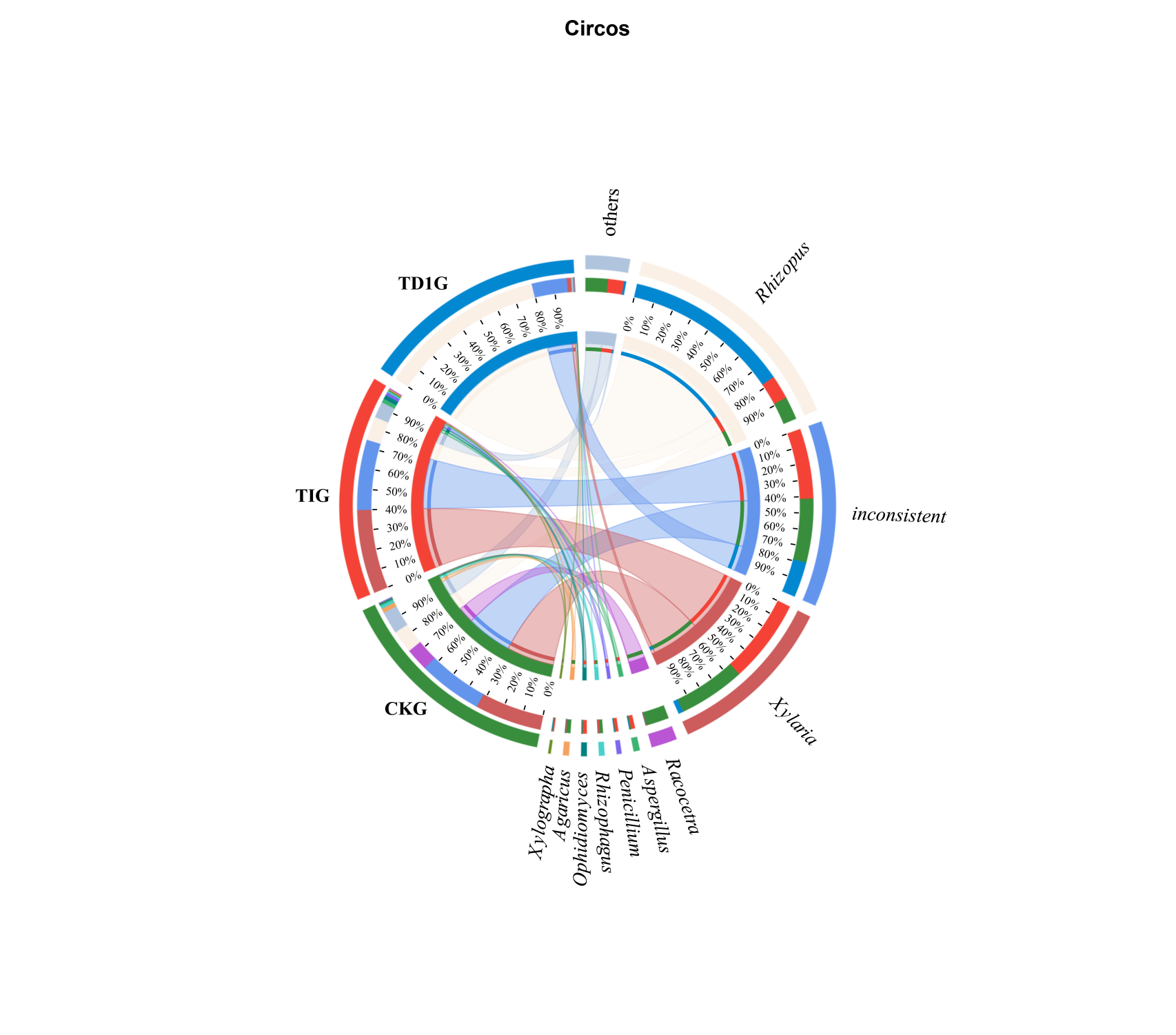


**Figure S6**. Fungal community composition in different samples. The left semicircle represents the species abundance composition of the sample (percentage), and the right semicircle represents the distribution of species in different samples at the genus level (percentage).

**

**

**Figure S7**. Variation patterns of pH in different AD systems (a). Effect of pH on bacterial communities, (b). Effect of pH on archaeal communities (c).


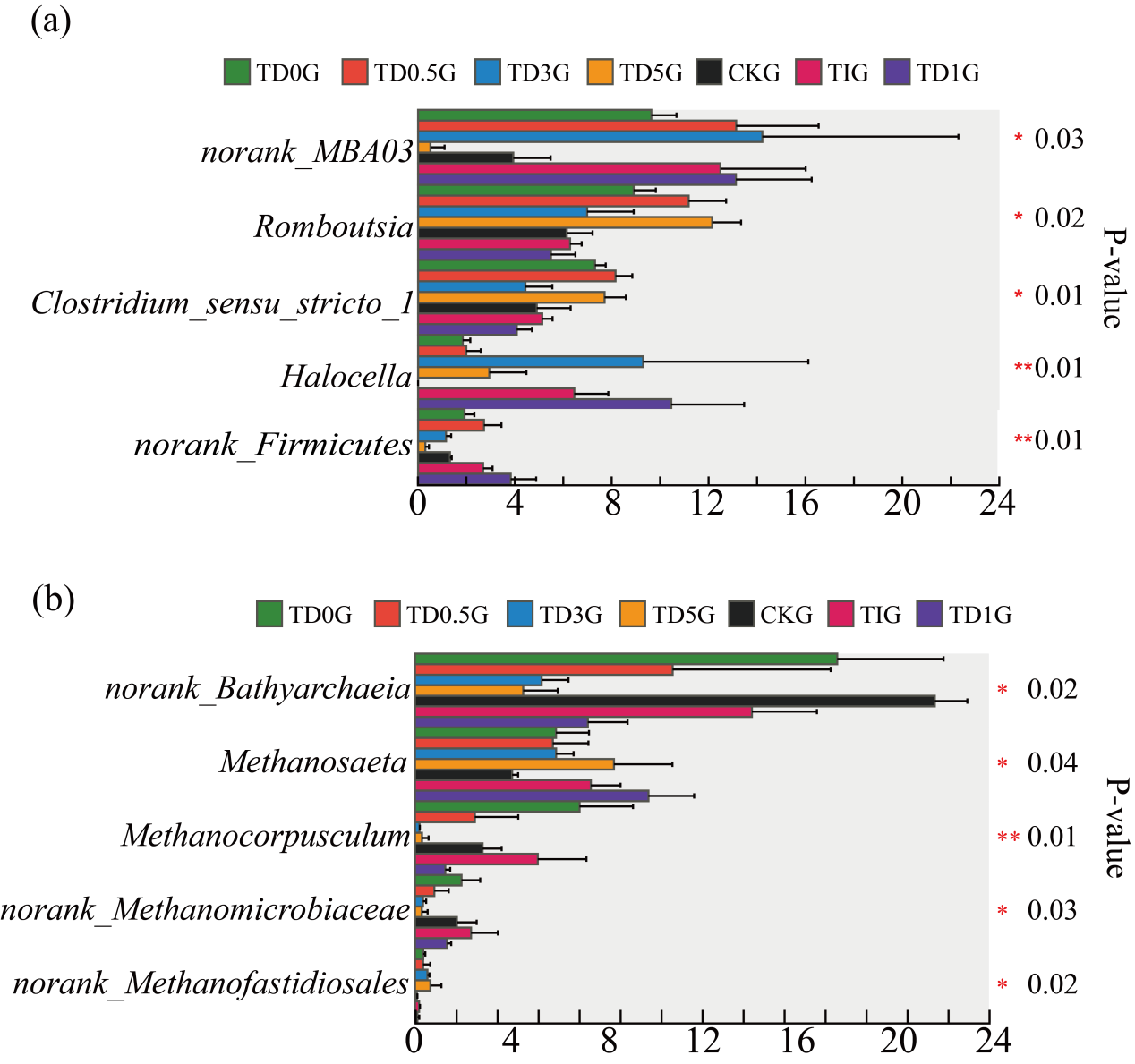


**Figure S8**. The bacteria (a) and archaea (b) with most significant differences in the abundance under different AD conditions.


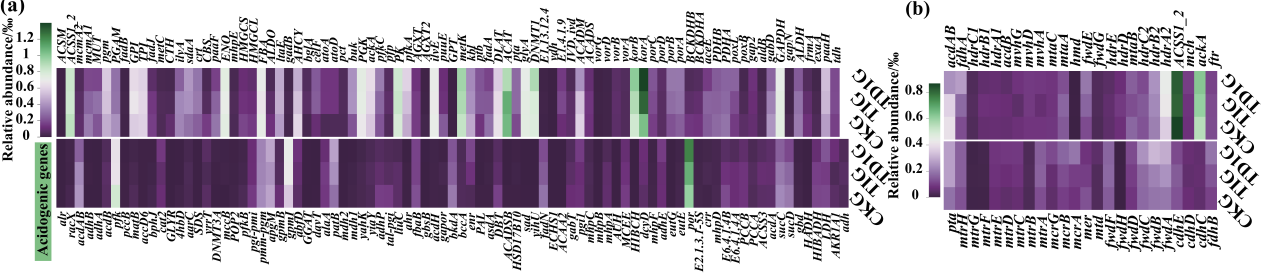


**Figure S9**. The relative abundance of genes involved in VFA production (a) and genes involved in CH_4_ production processes (b).


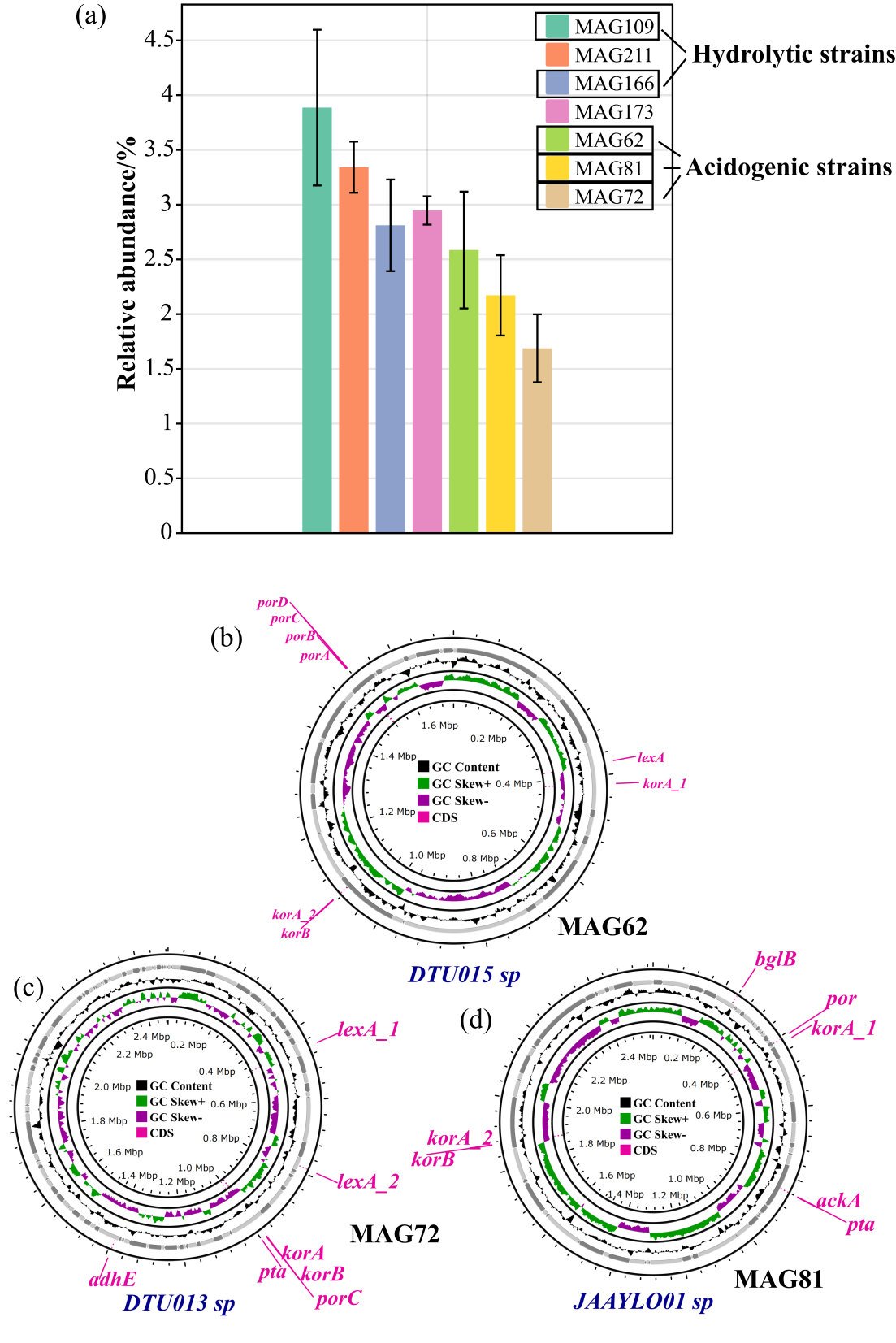


**Figure S10**. MAGs composition in TD1G samples based on metagenomic data (a). Circadian diagrams of the genomes of key strains *DTU015 sp.* and the key hydrolytic and acidogenic genes it carries (b). Circadian diagrams of the genomes of key strains *DTU013 sp.* and the key hydrolytic and acidogenic genes it carries (c). Circadian diagrams of the genomes of key strains *JAAYYLO01 sp.* and the key hydrolytic and acidogenic genes it carries (d).


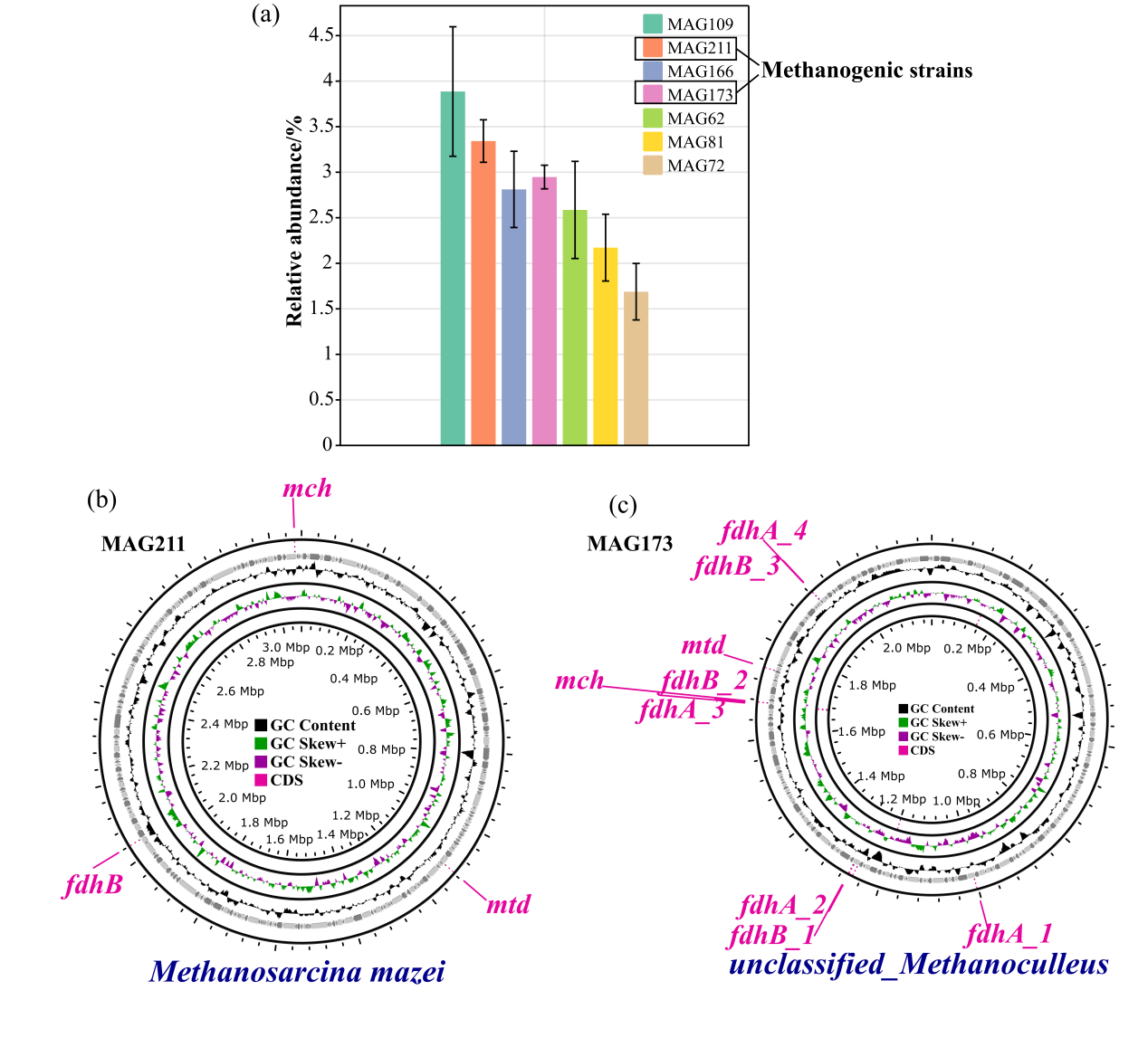


**Figure S11**. MAGs composition in TD1G samples based on metagenomic data (a). Circadian diagrams of the genomes of key strains *Methanosarcina_mazei* and the key genes it carries (b). Circadian diagrams of the genomes of key strains *unclassified*_*Methanoculleus* and the key hydrolytic and acidogenic genes it carries (c).

**
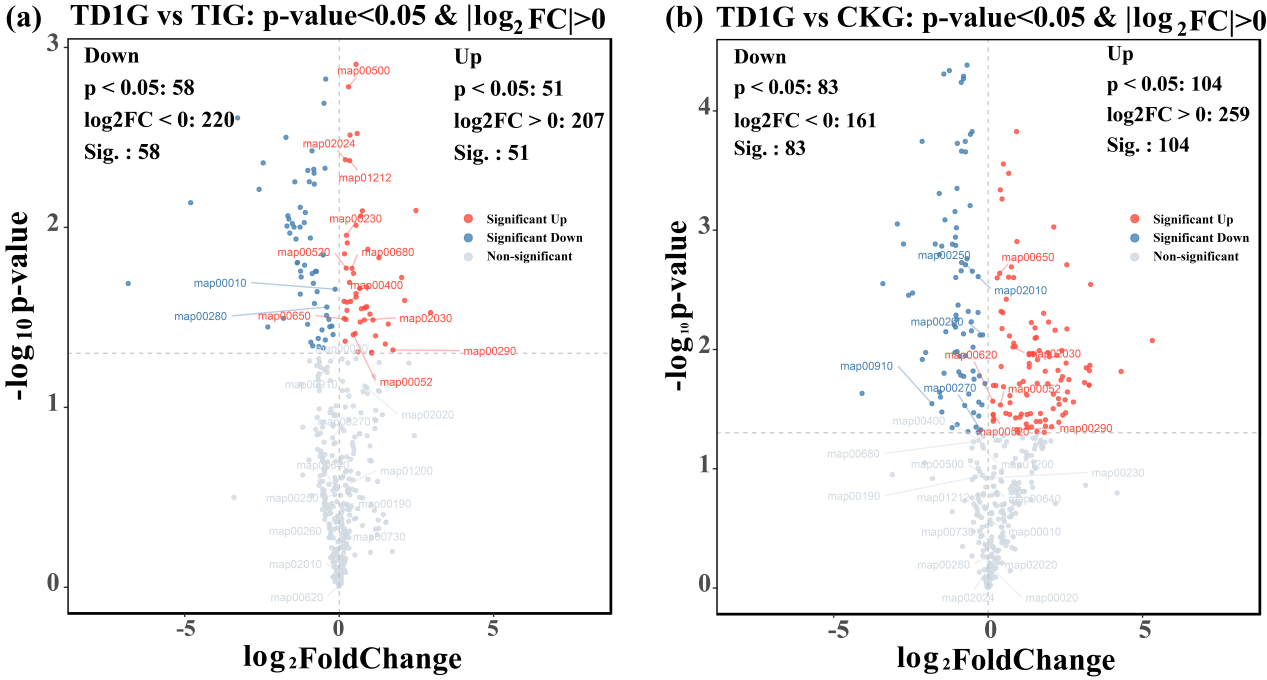
**

**Figure S12**. The expression level of high-abundance pathways in CKG (a). The expression level of high-abundance pathways in TIG (b). The expression level of high-abundance pathways in TD1G (c).


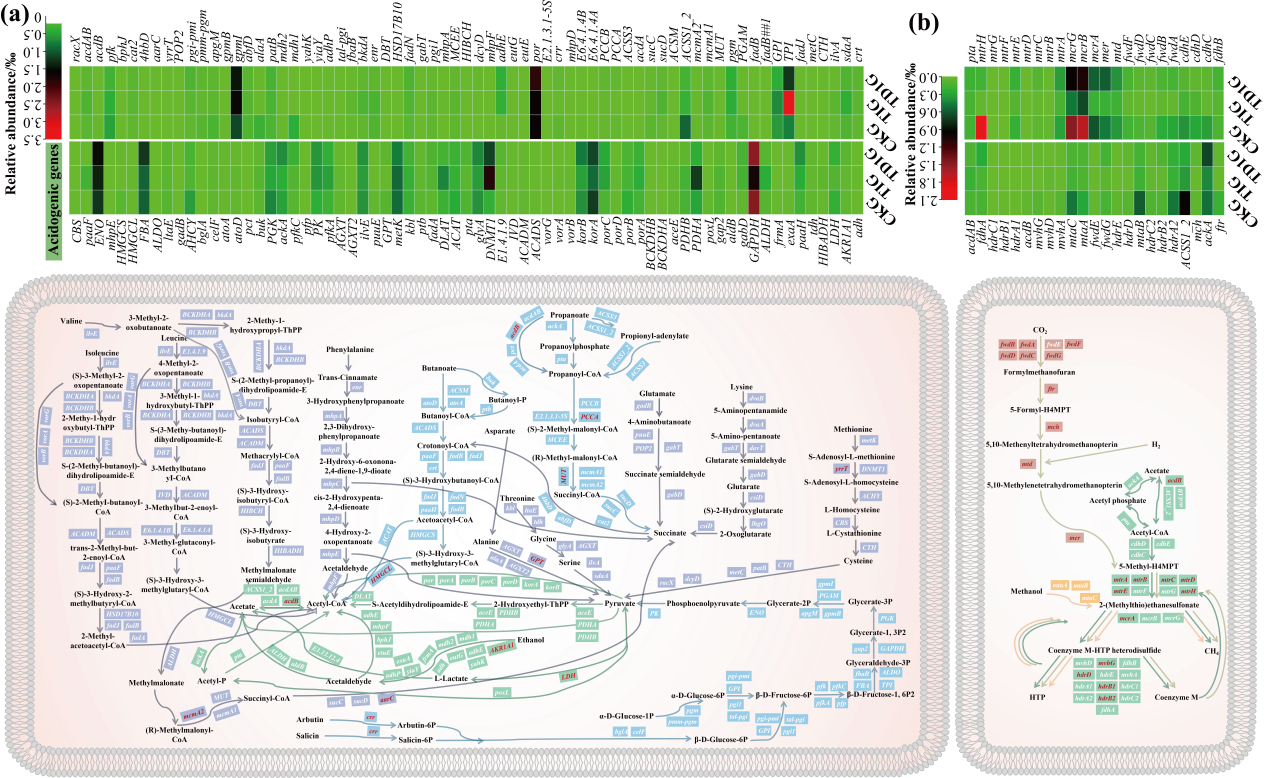


**Figure S13**. The expression level of acidogenic genes with related pathway (a) and expression level of methanogenic genes with the related acidogenic pathway (b). The red-labeled genes meant that the gene abundance in TD1G was significantly higher than that in TIG (log2FC＞1.0, P＜0.05).

**
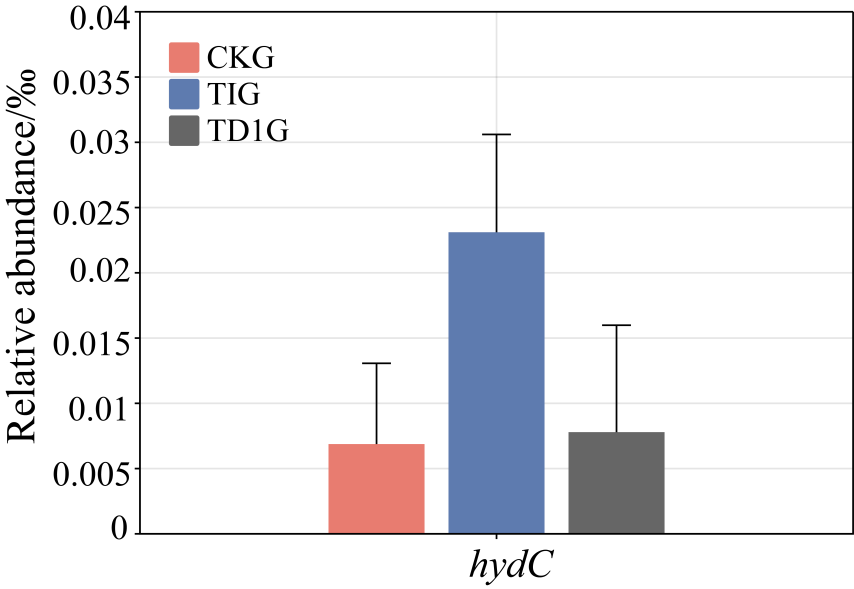
**

**Figure S14**. The expression level of genes *hydC* encoding Hase. These expression level of genes are not significant.

**
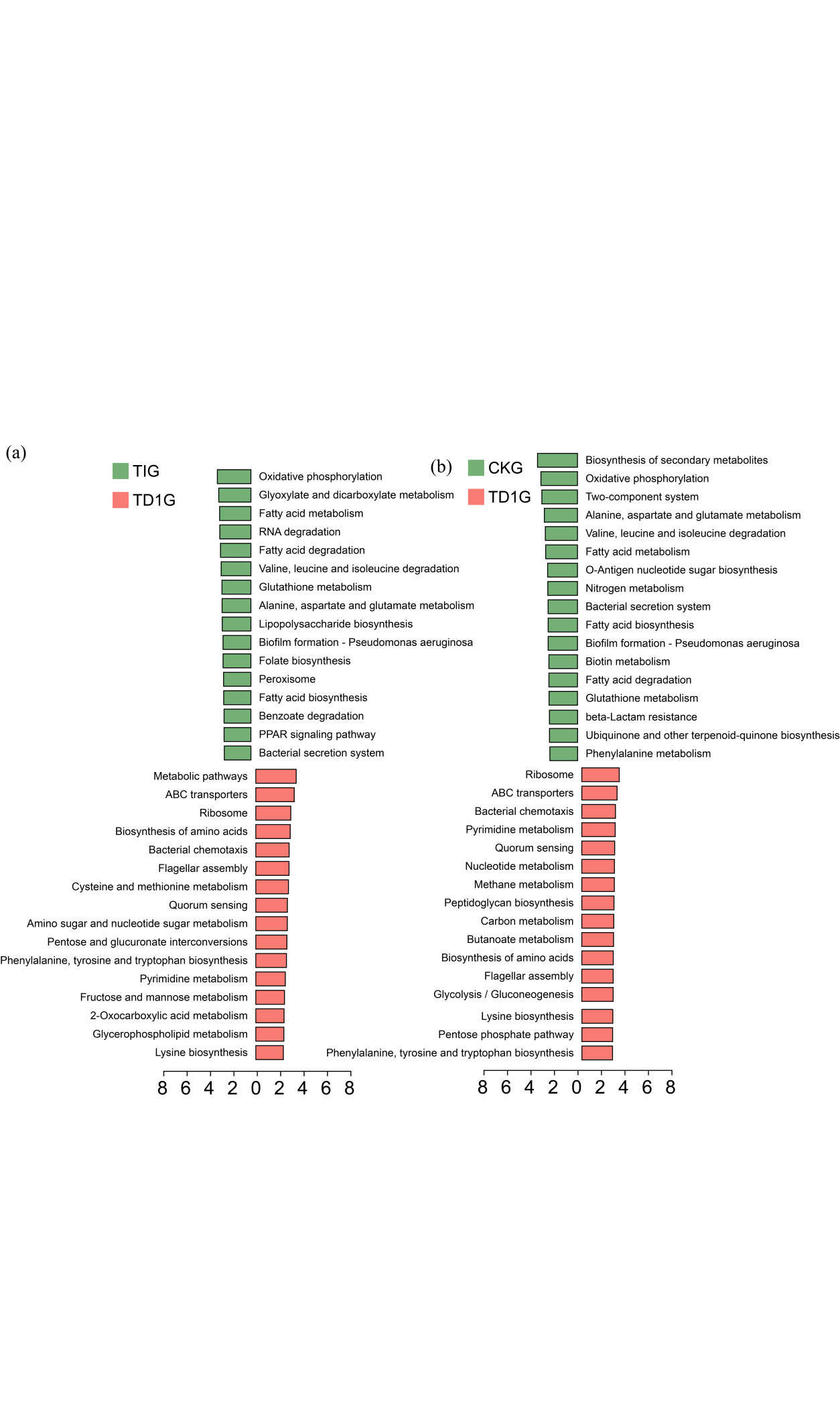
**

**Figure S15**. Linear discriminant analysis effect size (LEfSe) of the key pathways of the AD system after inoculation and hydrolysis pretreatment and the AD system inhibited by TAN (a) and LEfSe of the key pathways of the AD system after inoculation and hydrolysis pretreatment and the uninhibited AD system (b). LEfSe used linear discriminant analysis (LDA) to estimate the effect of the abundance of each genus on the difference of samples. LDA > 4 here.


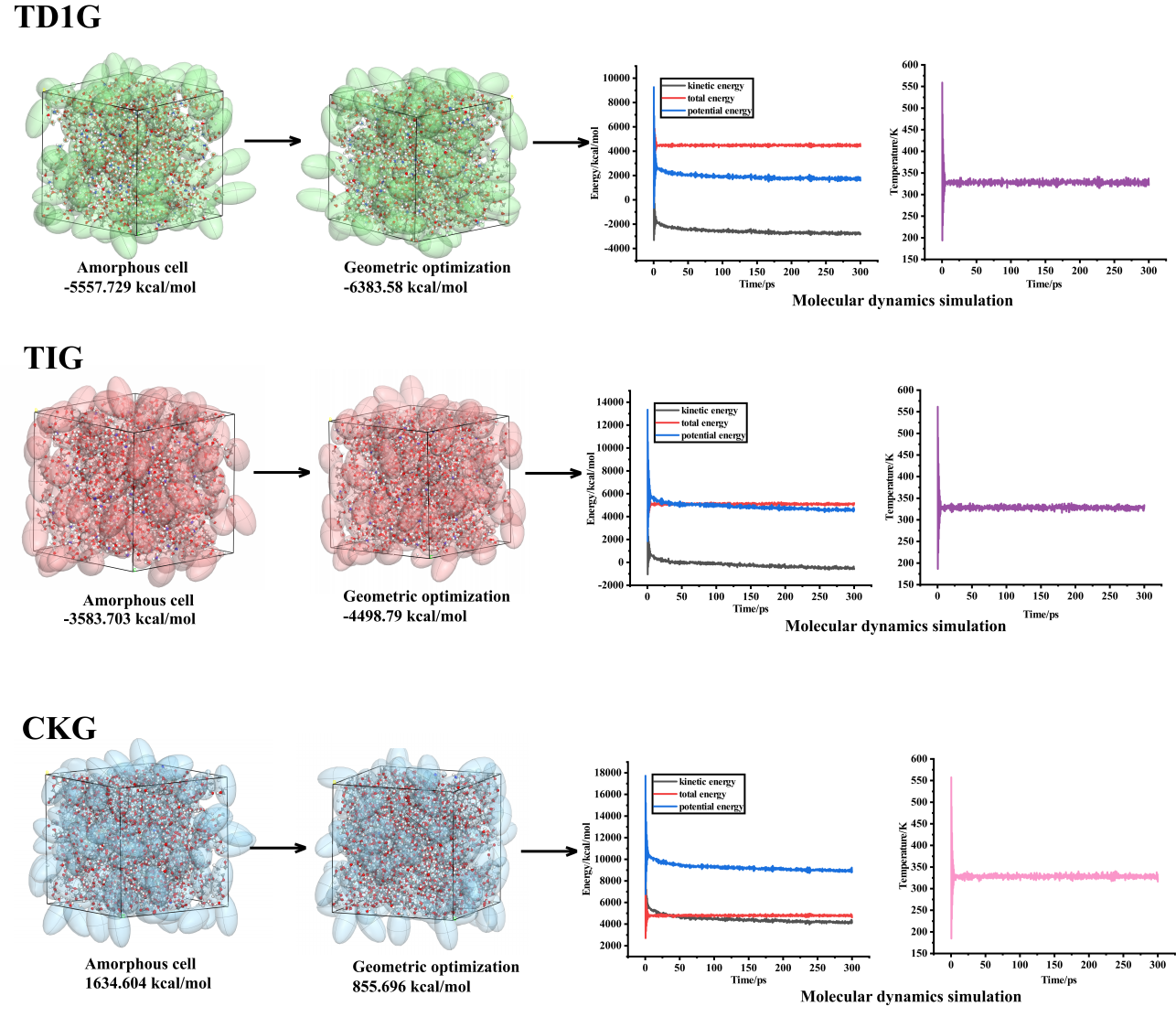


**Figure S16**. Molecular dynamics simulation process in different AD systems. According to the trend of energy curve, the number of frames of 20-300 ps is selected to calculate the diffusion coefficient.


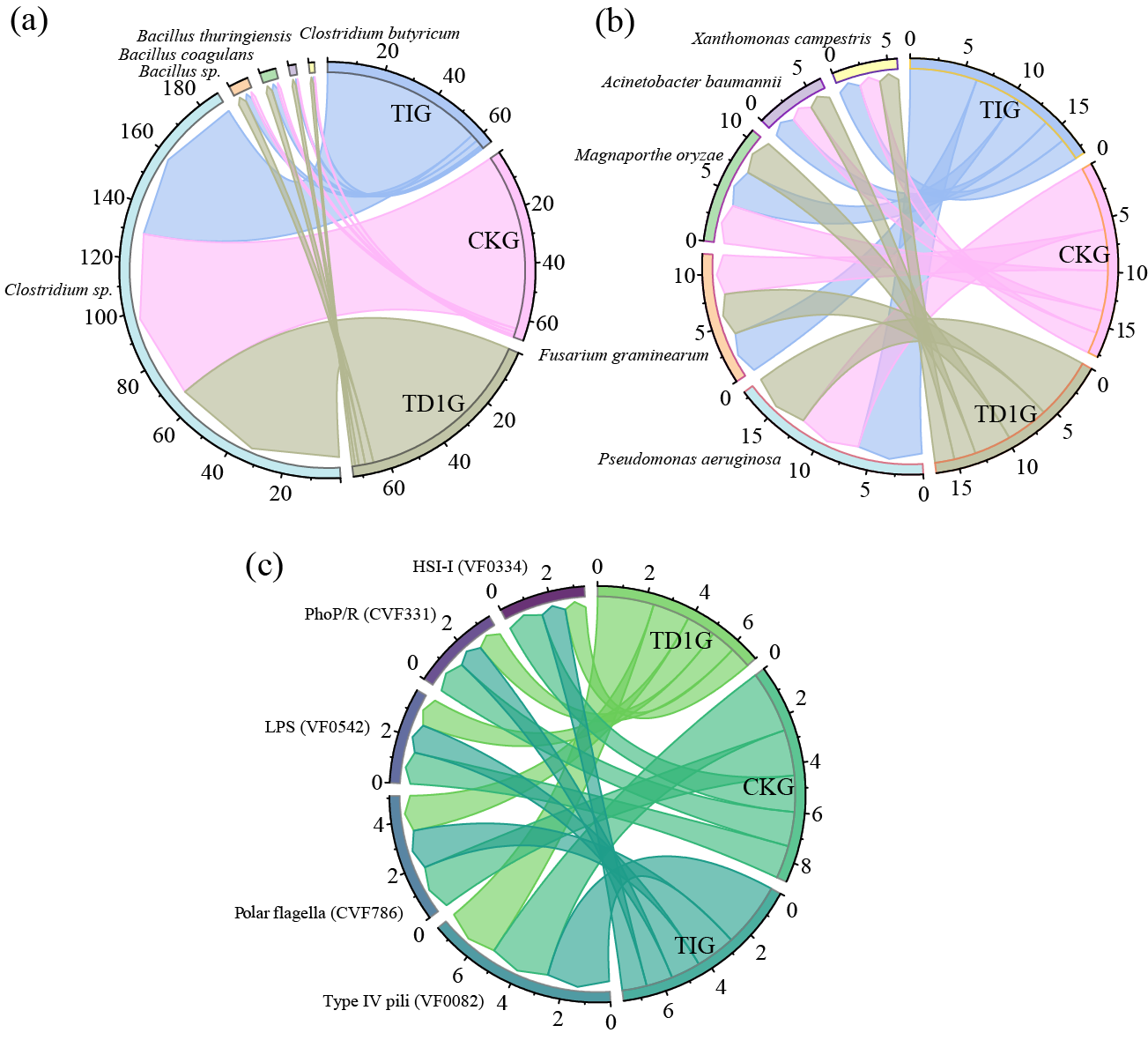


**Figure S17**. Probiotic abundance (a), pathogenic bacteria abundance (b) and virulence factor abundance (c).


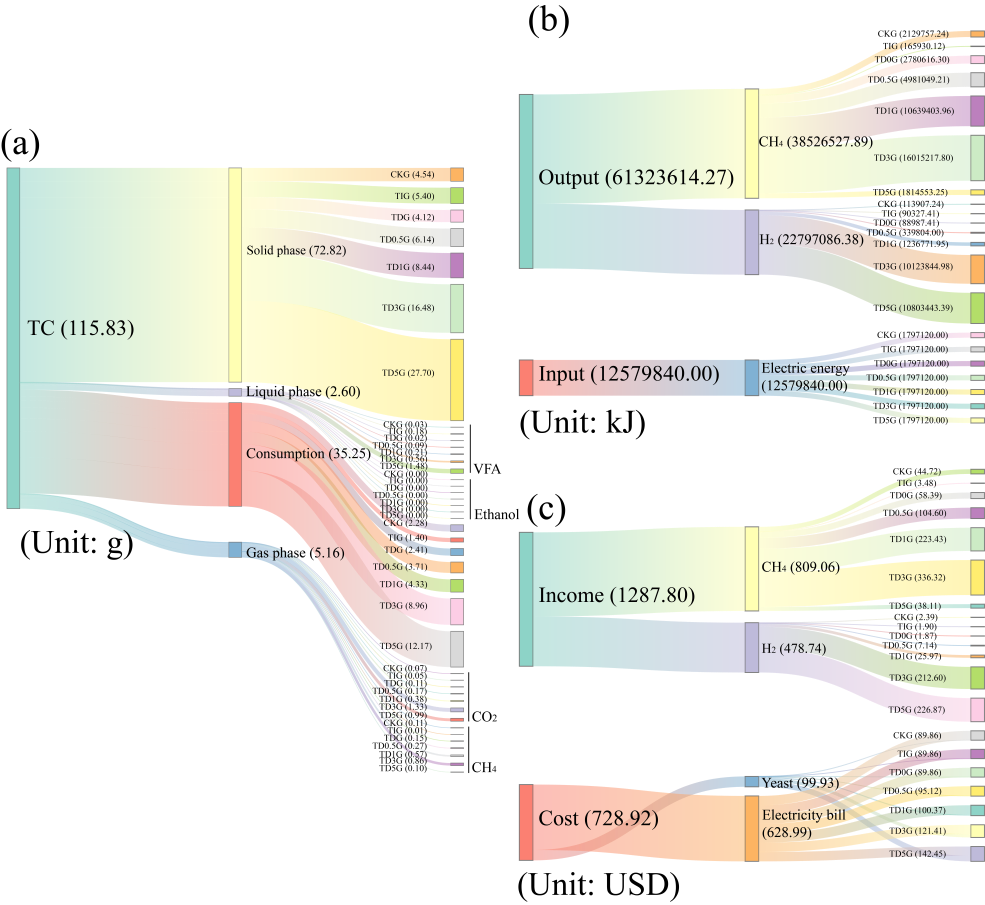


**Figure S18**. Carbon balance calculation (a), energy balance calculation (b), and balance of payments calculation (c).


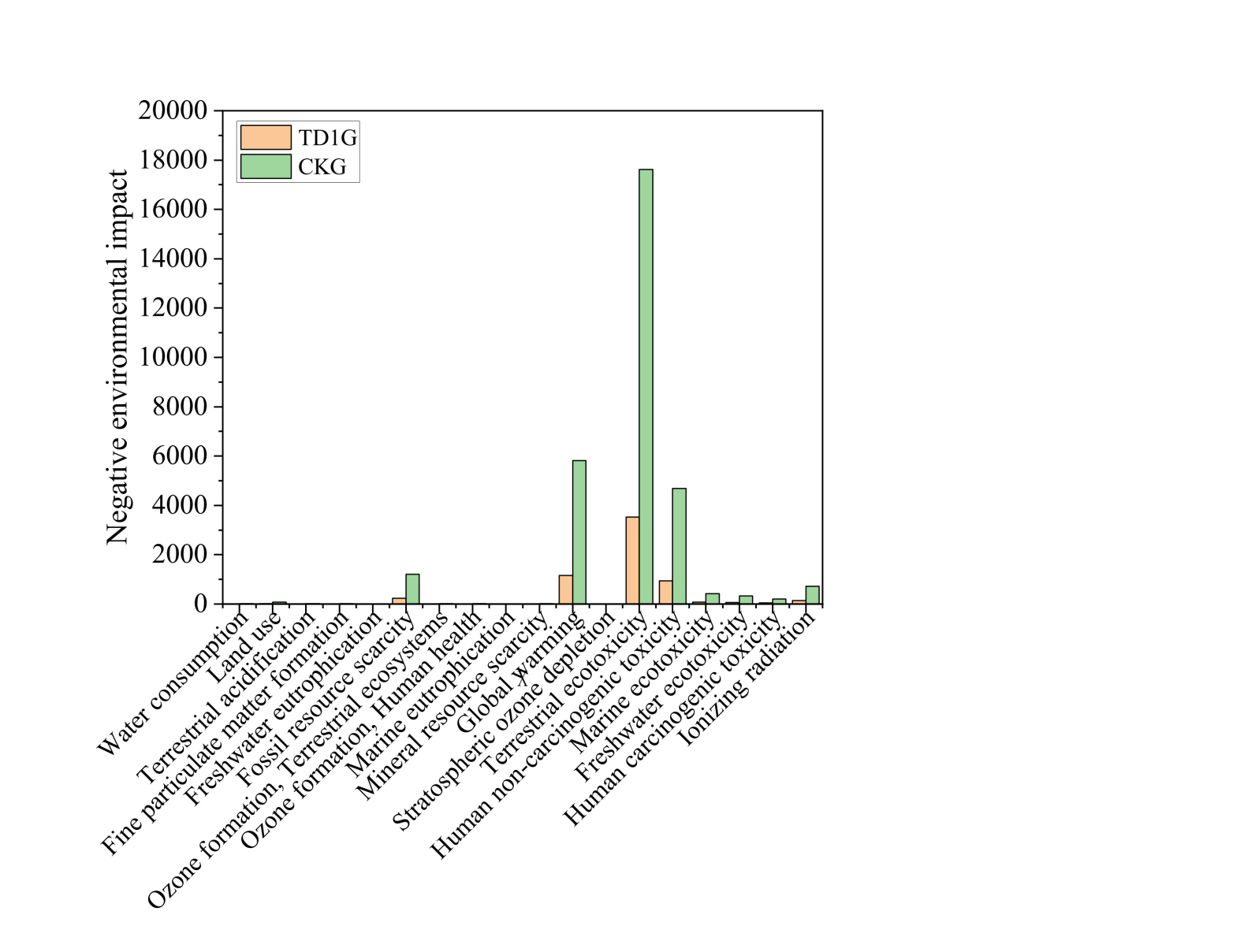


**Figure S19**. Comparison of the environmental negative impacts of TD1G and CKG based on the ReCiPe2016 analysis method


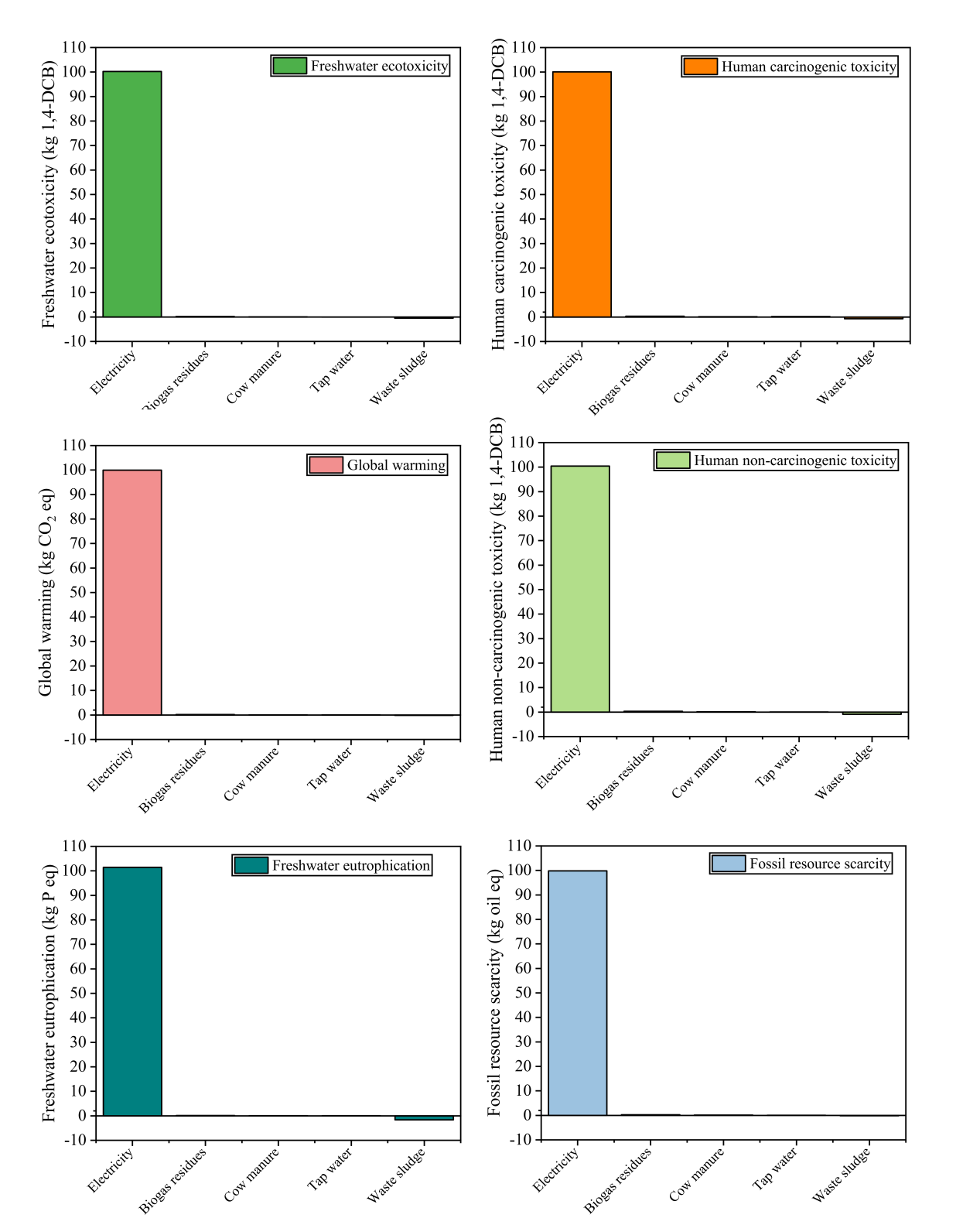


**Figure S20.** Contribution of various input substances to major environmental negative impacts in CKG


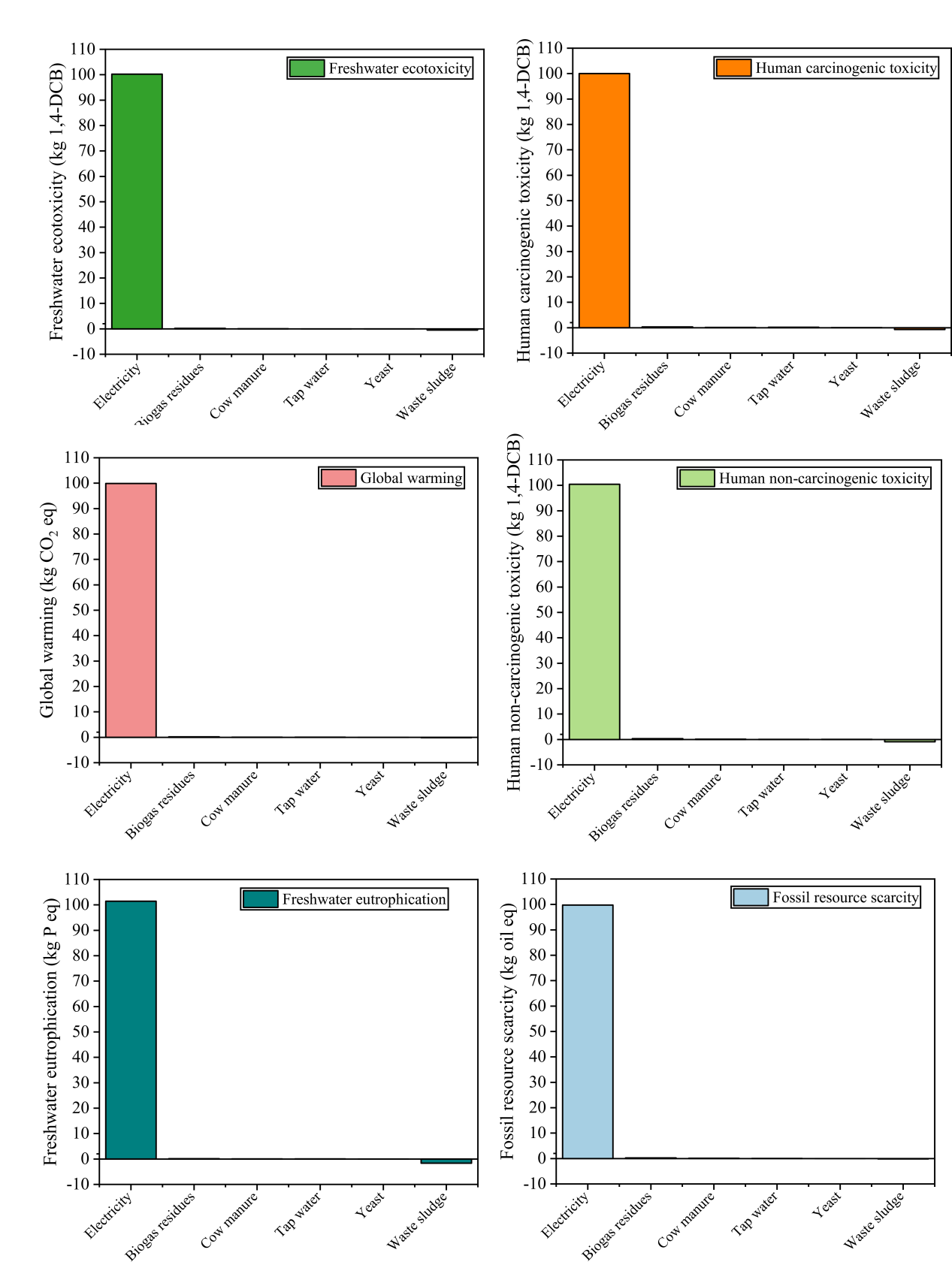


**Figure S21.** Contribution of various input substances to major environmental negative impacts in TD1G


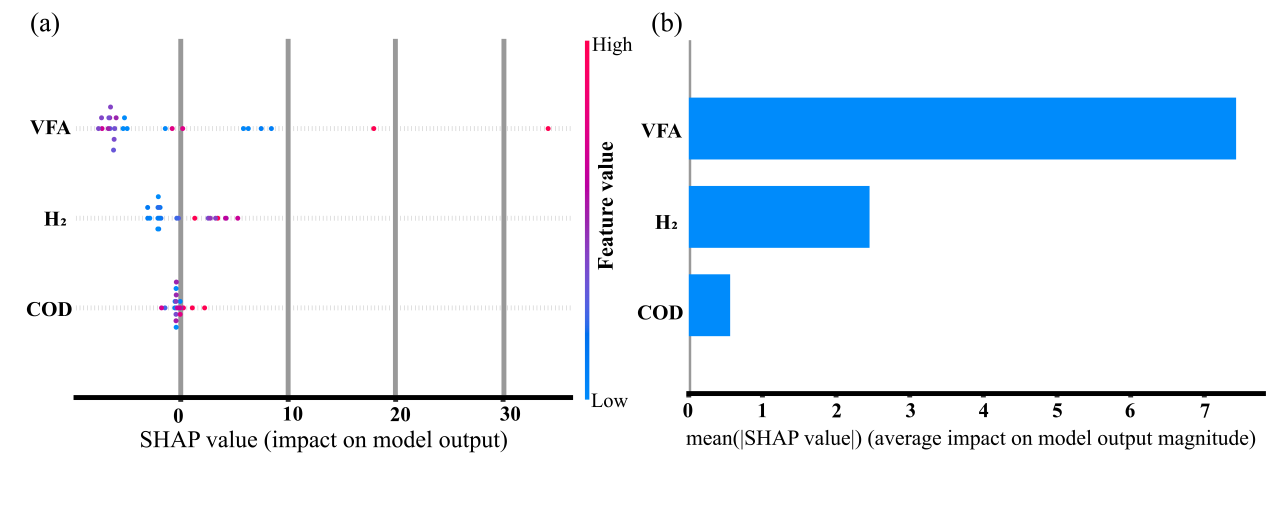


**Figure S22**. SHAP values (a), ranking of the importance of parameters to the model results (b).

**Machine Learning Methods**

This study utilizes the Random Forest model for analysis. The construction principles of the Random Forest are illustrated in Figure S23. Random Forest is based on individual decision trees, which offer a clear and comprehensible decision-making process while being less dependent on data distribution. This makes it particularly well-suited for handling real data with complex distributions. The combination of multiple decision trees effectively reduces the model's error rate, minimizing the risks of overfitting and underfitting, and enhancing generalization ability. The machine learning model was built using Python 3.7. The modeling process includes steps such as data preprocessing, model training and hyperparameter optimization, and model evaluation. The scripts and code can be found in the GitHub repository.

Specific details are as follows:

***Hardware Information***. The computer used is a LENOVO MT 82YA BU Idea FM Legion Y7000P IRH8, equipped with a 13th Gen Intel(R) Core(TM) i7-13620H CPU and running Windows 11 Pro (x64).

***Data Features***. The dataset comprises genetic data, microbial genus data, and AD performance. The dataset contains 279 groups, with data augmentation applied during the training process. The ratio of the training set to the prediction set is 4:1. The model training and hyperparameter optimization processes were completed using the training set, selecting CH_4_ production as the prediction target, with VFA, COD, and H_2_ as input features.

***Hyperparameter Optimization of the Random Forest Model***. GridSearchCV was used for hyperparameter tuning of the model. The main hyperparameters targeted for optimization include: n_estimators [100, 200, 300], max_depth [5, 10, 15], min_samples_split [2, 4, 6], and min_samples_leaf [1, 2, 3]. Adjusting parameters such as max_depth, min_samples_split, and min_samples_leaf helps control the complexity of the decision trees, preventing overfitting or underfitting. Tuning n_estimators strikes a balance between model performance and computational time.

***Model Performance Evaluation***. After training and hyperparameter optimization, the model was tested on the test set to evaluate its accuracy. Since the model constructed is a regression prediction model for continuous variables, the coefficient of determination (R²), root mean square error (RMSE), and mean squared error (MSE) were chosen to assess the model’s predictive performance and accuracy.

**Figure S23.** Framework for constructing the random forest model

**Life Cycle Assessment Method**

***Research Subjects.*** Life Cycle Assessment (LCA) is suitable for environmental impact analysis and can comprehensively and intuitively evaluate the negative environmental impacts of the AD process. It's important to note that the TIG process almost fails to produce CH_4_, so it is assumed to have the greatest negative impact on the environment and is not included in the LCA analysis. This study only conducts LCA analyses on TD1G and CKG, examining and comparing the negative environmental impacts associated with the production of 1 m³of CH_4_ from both processes. The specific system boundaries are shown in Figure S24. The focus of this study is on comparing the negative environmental impacts of TD1G and CKG; therefore, a “cradle-to-gate” approach is adopted, without considering the environmental impacts of subsequent CH_4_ power generation processes.

**Figure S24.** The system boundary of LCA for AD

***Inventory Analysis.*** The LCA inventory summarizes the inputs and outputs related to the functional unit throughout the life cycle. The LCA inventory is constructed using the experimental data from this study, as shown in Table S11. Upstream processes are automatically linked to facilitate a comprehensive assessment of the environmental impacts of AD. Use software OpenLCA (version 2.3.1) for assessment. The databases used are mainly the Tiangong database and the ecoinvent database.

**Table S11**. LCA Inventory

|  | Value-TD1G | Value-CKG | Unit | Stages |
| --- | --- | --- | --- | --- |
| Waste sludge | 34.78 | 577.66 | kg | Raw material acquisition |
| Cow manure | 115.63 | 4.17 | kg |  |
| Yeast | 18.26 | 0 | kg |  |
| Tap water | 153.89 | 768.76 | kg |  |
| Electricity | 1736.05 | 8672.69 | kwh | CH_4_ production |
| Biogas residues | 273.00 | 1363.79 | kg | Waste utilization |

Note: All elements in Table S1 have accounted for the corresponding transportation processes.

***Evaluation Analysis.*** The LCA inventory data alone cannot directly quantify the extent of environmental impacts from the AD process. Establishing a model is necessary to convert the inventory data into quantitative environmental loads, reflecting specific levels of environmental impact. ReCiPe 2016 is a widely used method for environmental impact assessment that allows for a detailed evaluation of environmental impacts based on 18 midpoint indicators (such as terrestrial ecotoxicity, global warming, human non-carcinogenic toxicity, fossil resource scarcity, and ionizing radiation) [7]. This study employs the ReCiPe 2016 method to assess the environmental impacts of the TD1G and CKG processes.

**References**

1. Kor-Bicakci, G. et al. Effect of dewatered sludge microwave pretreatment temperature and duration on net energy generation and biosolids quality from anaerobic digestion. ENERGY 168, 782-795 (2019).

2. Zhao, S.N. et al. Anaerobic co-digestion of chicken manure and cardboard waste: Focusing on methane production, microbial community analysis and energy evaluation. BIORESOURCE TECHNOLOGY 321 (2021).

3. Kang D D, et al. MetaBAT, an efficient tool for accurately reconstructing single genomes from complex microbial communities. PEERJ 3(8), 1165 (2015).

4. Sieber C M K, et al. Recovery of genomes from metagenomes via a dereplication, aggregation and scoring strategy. NATURE MICROBIOLOGY 3(7), 836 (2018).

5. Parks D H, et al. CheckM: assessing the quality of microbial genomes recovered from isolates, single cells, and metagenomes. GENOME RESEARCH 25(7), 1043 (2015).

6. Parks D H, et al. GTDB: an ongoing census of bacterial and archaeal diversity through a phylogenetically consistent, rank normalized and complete genome-based taxonomy. NUCLEIC ACIDS RESEARCH 50 (D1), D785-D794 (2022).

7. Mark A. J. et al. ReCiPe2016: a harmonised life cycle impact assessment method at midpoint and endpoint level. THE INTERNATIONAL JOURNAL OF LIFE CYCLE ASSESSMENT, 2016, 22(2): 138-147.
